# Search for domain wall dark matter with atomic clocks on board global positioning system satellites

**DOI:** 10.1038/s41467-017-01440-4

**Published:** 2017-10-30

**Authors:** Benjamin M. Roberts, Geoffrey Blewitt, Conner Dailey, Mac Murphy, Maxim Pospelov, Alex Rollings, Jeff Sherman, Wyatt Williams, Andrei Derevianko

**Affiliations:** 1 Department of Physics, University of Nevada, Reno, NV 89557 USA; 2 Nevada Geodetic Laboratory, Nevada Bureau of Mines and Geology, University of Nevada, Reno, NV 89557 USA; 3 Department of Physics and Astronomy, University of Victoria, Victoria, BC Canada V8P 1A1; 4 Perimeter Institute for Theoretical Physics, Waterloo, ON Canada N2J 2W9; 5 National Institute of Standards and Technology, Boulder, CO 80305 USA

## Abstract

Cosmological observations indicate that dark matter makes up 85% of all matter in the universe yet its microscopic composition remains a mystery. Dark matter could arise from ultralight quantum fields that form macroscopic objects. Here we use the global positioning system as a ~ 50,000 km aperture dark matter detector to search for such objects in the form of domain walls. Global positioning system navigation relies on precision timing signals furnished by atomic clocks. As the Earth moves through the galactic dark matter halo, interactions with domain walls could cause a sequence of atomic clock perturbations that propagate through the satellite constellation at galactic velocities ~ 300 km s^−1^. Mining 16 years of archival data, we find no evidence for domain walls at our current sensitivity level. This improves the limits on certain quadratic scalar couplings of domain wall dark matter to standard model particles by several orders of magnitude.

## Abstract (web summary)

The composition of dark matter in the universe remains a mystery, with one hypothetical form being topological defects. Here the authors determine a stronger constraint on the coupling of this dark matter to atomic clocks on board global positioning satellites through the analysis of 16 years of archival data.

## Introduction

Despite the overwhelming cosmological evidence for the existence of dark matter (DM), there is as of yet no definitive evidence for DM in terrestrial experiments. Multiple cosmological observations suggest that ordinary matter makes up only about 15% of the total matter in the universe, with the remaining portion composed of DM^1^. All the evidence for DM (e.g., galactic rotation curves, gravitational lensing, cosmic microwave background) comes from galactic or larger scale observations through the gravitational pull of DM on ordinary matter^1^. Extrapolation from the galactic to laboratory scales presents a challenge because of the unknown nature of DM constituents. Various theories postulate additional non-gravitational interactions between standard model (SM) particles and DM. Ambitious programs in particle physics have mostly focused on (so far unsuccessful) searches for weakly interacting massive particle (WIMP) DM candidates with 10–10^3^ GeV *c* ^−2^ masses (*c* is the speed of light) through their energy deposition in particle detectors^2^. The null results of the WIMP searches have partially motivated an increased interest in alternative DM candidates, such as ultralight fields. These fields, in contrast to particle candidates, act as coherent entities on the scale of an individual detector.

Here we focus on ultralight fields that may cause apparent variations in the fundamental constants of nature. Such variations in turn lead to shifts in atomic energy levels, which may be measurable by monitoring atomic frequencies^3–5^. Such monitoring is performed naturally in atomic clocks, which tell time by locking the frequency of externally generated electromagnetic radiation to atomic frequencies. Here, we analyse time as measured by atomic clocks on board global positioning system (GPS) satellites to search for DM-induced transient variations of fundamental constants^6^. In effect we use the GPS constellation as a ~ 50,000 km-aperture DM detector. Our DM search is one example of using GPS for fundamental physics research. Another recent example includes placing limits on gravitational waves^7^.

GPS works by broadcasting microwave signals from nominally 32 satellites in medium-Earth orbit. The signals are driven by an atomic clock (either based on Rb or Cs atoms) on board each satellite. By measuring the carrier phases of these signals with a global network of specialised GPS receivers, the geodetic community can position stations at the 1 mm level for purposes of investigating plate tectonics and geodynamics^8^. As part of this data processing, the time differences between satellite and station clocks are determined with <0.1 ns accuracy^9^. Such high-quality timing data for at least the past decade are publicly available and are routinely updated. Here we analyse data from the Jet Propulsion Laboratory^10^. A more detailed overview of the GPS architecture and data processing relevant to our search is given in Supplementary Note 1, which includes Supplementary Figs. 1 and 2 and Supplementary Table 1.

The large aperture of the GPS network is well suited to search for macroscopic DM objects, or clumps. Examples of clumpy DM candidates are numerous: topological defects (TDs)^11,12^, *Q*-balls^13–15^, solitons^16,17^, axion stars^18,19^ and other stable objects formed due to dissipative interactions in the DM sector. For concreteness, we consider specifically TDs. Each TD type (monopoles, strings or domain walls) would exhibit a transient in GPS data with a distinct signature.

Topological defects may be formed during the cooling of the early universe through a spontaneous symmetry breaking phase transition^11,12^. Technically, this requires the existence of hypothesised self-interacting DM fields, *φ*. While the exact nature of TDs is model-dependent, the spatial scale of the DM object, *d*, is generically given by the Compton wavelength of the particles that make up the DM field $d = \hbar /(m_\varphi c)$, where *m*_*φ*_ is the field particle mass, and $\hbar$ is the reduced Plank constant. The fields that are of interest here are ultralight: for an Earth-sized object the mass scale is $m_\varphi \sim 10^{ - 14}{\kern 1pt} {\mathrm{eV}}\,c^{ - 2}$, hence the probed parameter space is complementary to that of WIMP searches^2^, as well as searches for other DM candidates^20–22^. Searches for TDs have been performed via their gravitational effects, including gravitational lensing^23–25^. Limits on TDs have been placed by the Planck^26^ and Background Imaging of Cosmic Extragalactic Polarization 2 (BICEP2)^27^ collaborations from fluctuations in the cosmic microwave background. So far the existence of TDs is neither confirmed nor ruled out. The past few years have brought several proposals for TD searches via their non-gravitational signatures^6,28–32^.

Here, we report the results of the search for domain walls, quasi-2D cosmic structures. As a result, we improve the limits on certain quadratic scalar couplings of domain wall DM to standard model particles by several orders of magnitude.

## Results

### Domain wall theory and expected signal

We focus on the search for domain walls, since they would leave the simplest DM signature in the data. General signature matching for the vast set of GPS data has proven to be computationally expensive and is in progress. While we interpret our results in terms of domain wall DM, we remark that our search applies equally to the situation where walls are closed on themselves, forming a bubble that has transverse size significantly exceeding the terrestrial scale. The galactic structure formation in that case may occur as per conventional cold dark matter theory^33^, since from the large distance perspective the bubbles of domain walls behave as point-like objects.

**Fig. 1:**
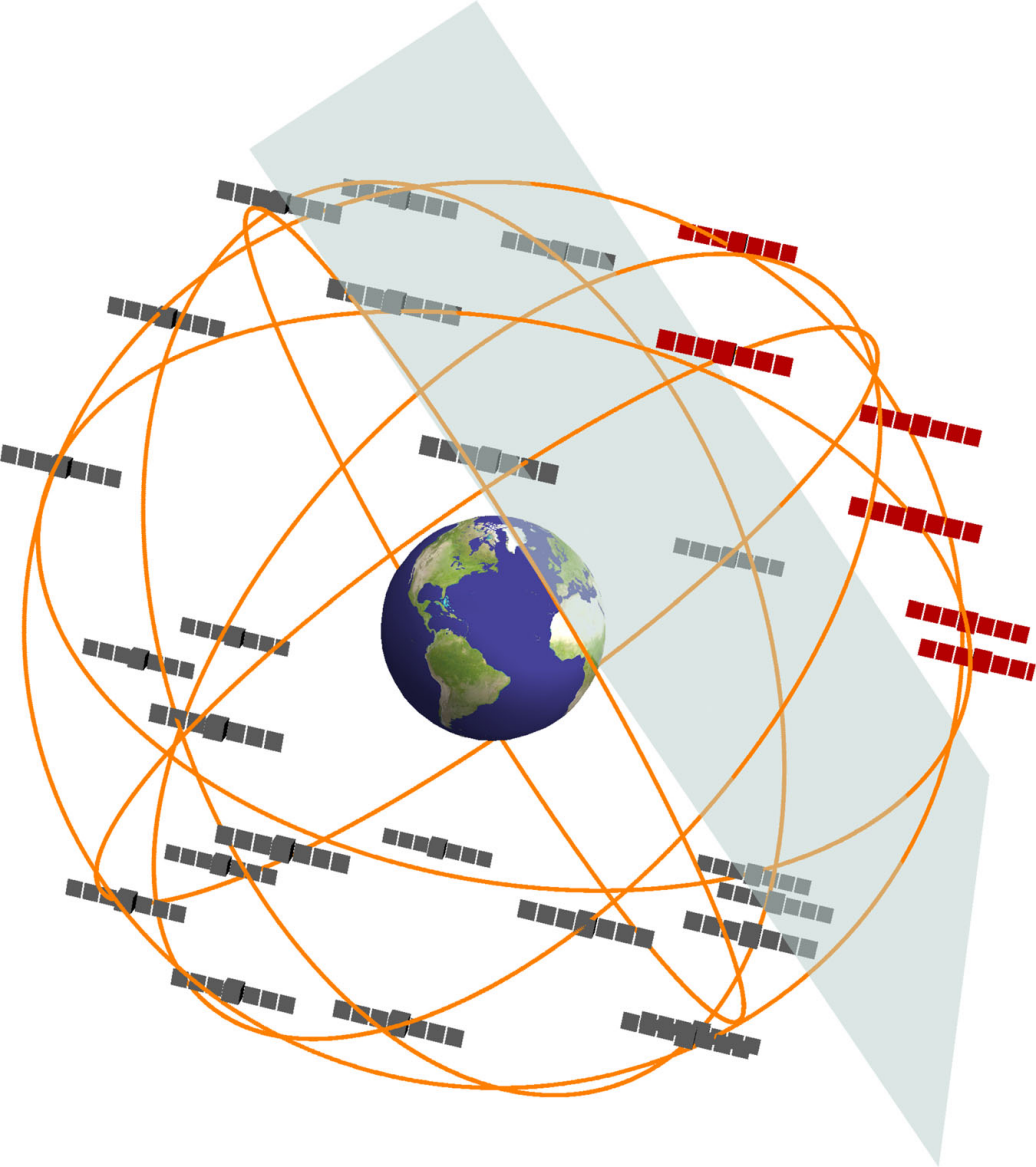
Domain wall crossing. As a domain wall sweeps through the Global Positioning System constellation at galactic velocities, *v*_*g*_ ~ 300 km s^−1^, it perturbs the atomic clocks on board the satellites causing a correlated propagation of glitches through the network. The red satellites have interacted with the domain wall, and exhibit a timing bias compared with the grey satellites. Image generated using Mathematica software^48^

We employ the known properties of the DM halo to model the statistics of encounters of the Earth with TDs. Direct measurements^34^ of the local dark matter density give 0.3 ± 0.1 GeV cm^−3^, and we adopt the value of *ρ*_DM_ ≈ 0.4 GeV cm^−3^ for definitiveness. According to the standard halo model, in the galactic rest frame the velocity distribution of DM objects is isotropic and quasi-Maxwellian, with dispersion^35^ $v \simeq 290$ km s^−1^ and a cut-off above the galactic escape velocity of $v_{{\mathrm{esc}}} \simeq 550$ km s^−1^. The Milky Way rotates through the DM halo with the Sun moving at ~ 220 km s^−1^ towards the Cygnus constellation. For the goals of this work we can neglect the much smaller orbital velocities of the Earth around the Sun (~ 30 km s^−1^) and GPS satellites around the Earth (~ 4 km s^−1^). Thereby one may think of a TD wind impinging upon the Earth, with typical relative velocities $v_g\sim 300$ km s^−1^. Assuming the standard halo model, the vast majority of events (~ 95%) would come from the forward-facing hemisphere centred about the direction of the Earth’s motion through the galaxy, with typical transit times through the GPS constellation of about 3 min. An example of a domain wall crossing is shown in Fig. 1. Note that we make an additional assumption that the distribution of wall velocities is similar to the standard halo model, which is expected if the gravitational force is the main force governing wall dynamics within the galaxy. However, even if this distribution is somewhat different, the qualitative feature of a TD wind is not expected to change.

**Fig. 2:**
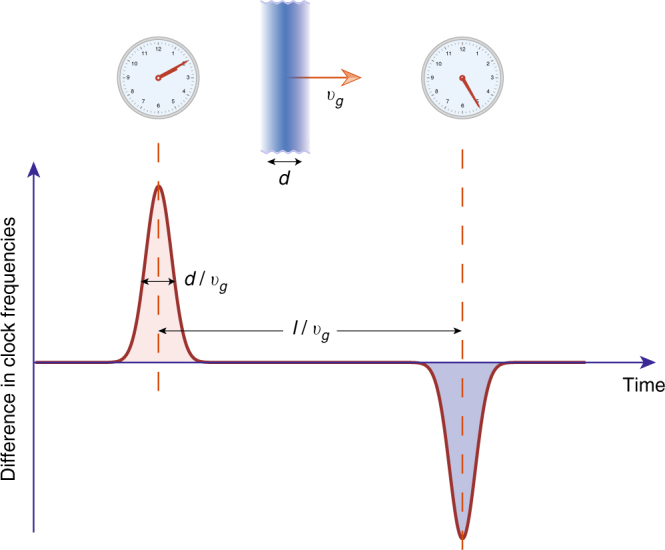
Time dependence of the dark matter-induced signal. The frequency difference between two identical ideal clocks separated by distance *l*. The time delay in the signals encodes the kinematics of the dark matter object

A positive DM signal can be visualised as a coordinated propagation of clock glitches at galactic velocities through the GPS constellation, see Fig. 2. The powerful advantage of working with the network is that non-DM clock perturbations do not mimic this signature. The only systematic effect that has propagation velocities comparable to *v*_*g*_ is the solar wind^36^, an effect that is simple to exclude based on the distinct directionality from the Sun and the fact that the solar wind does not affect the satellites in the Earth’s shadow.

As the nature of non-gravitational interactions of DM with ordinary matter is unknown, we take a phenomenological approach that respects the Lorentz and local gauge invariances. We consider quadratic scalar interactions between the DM objects and clock atoms that can be parameterised in terms of shifts in the effective values of fundamental constants^6^. The relevant combinations of fundamental constants include $\alpha = e^2/\hbar c \approx 1/137$, the dimensionless electromagnetic fine-structure constant (*e* is the elementary charge), the ratio *m*_*q*_/*Λ*_QCD_ of the light quark mass to the quantum chromodynamics (QCD) energy-scale, and *m*_*e*_ and *m*_*p*_, the electron and proton masses. With the quadratic scalar coupling, the relative change in the local value for each such fundamental constant is proportional to the square of the DM field

1 $$\frac{{\delta X\left( {{\mathbf{r}},t} \right)}}{X} = \Gamma _X\,\varphi \left( {{\mathbf{r}},t} \right)^2,$$

where *Γ*_*X*_ is the coupling constant between dark and ordinary matter, with *X* = *α*,*m*_*e*_,*m*_*p*_,*m*_*q*_/*Λ*_QCD_ (see Supplementary Note 2 for further details).

As the DM field vanishes outside the TD, the apparent variations in the fundamental constants occur only when the TD overlaps with the clock. This temporary shift in the fundamental constants leads in-turn to a transient shift in the atomic energy levels referenced by the clocks, which may be measurable by monitoring atomic frequencies^3–5^. The frequency shift can be expressed as

2 $$\frac{{\delta \omega \left( {{\mathbf{r}},t} \right)}}{{\omega _c}} = \mathop {\sum}\limits_X {K_X} \frac{{\delta X\left( {{\mathbf{r}},t} \right)}}{X},$$

where *ω*_*c*_ is the unperturbed clock frequency and *K*_*X*_ are known coefficients of sensitivity to effective changes in the constant *X* for a particular clock transition^37^. It is worth noting that the values of the sensitivity coefficients *K*_*X*_ depend on experimental realisation. Here we compare spatially separated clocks (to be contrasted with the conventional frequency ratio comparisons^3–5^), and thus our used values of *K*_*X*_ somewhat differ from other places in the literature^37^; full details are presented in Supplementary Note 2. For example, for the microwave frequency ^87^Rb clocks on board the GPS satellites, the sensitivity coefficients are

3 $$\frac{{\delta \omega }}{{\omega _c}}\left( {{\mathrm{Rb}}} \right) = \left( {4.34\,{\it{\Gamma }}_\alpha - 0.019\,{\it{\Gamma }}_q + {\it{\Gamma }}_{e/p}} \right)\,\varphi ^2 \equiv {\it{\Gamma }}_{{\mathrm{eff}}}^{\left( {{\mathrm{Rb}}} \right)}\,\varphi ^2,$$

where we have introduced the short-hand notation ${\it{\Gamma }}_q \equiv {\it{\Gamma }}_{m_q/\Lambda _{{\mathrm{QCD}}}}$ and ${\it{\Gamma }}_{e/p} \equiv 2{\it{\Gamma }}_{m_e} - {\it{\Gamma }}_{m_p}$, and the effective coupling constant *Γ*_eff_ ≡ ∑_*X*_ *K*_*X*_ *Γ*_*X*_.

From Eqs. (1) and (2), the extreme TD-induced frequency excursion, *δω*_ext_, is related to the field amplitude *φ*_max_ inside the defect as $\delta \omega _{{\mathrm{ext}}} = {\it{\Gamma }}_{{\mathrm{eff}}}\omega _c\varphi _{{\mathrm{max}}}^2$. Further, assuming that a particular TD type saturates the DM energy density, we have^6^ $\varphi _{{\mathrm{max}}}^2 = \hbar c\rho _{{\mathrm{DM}}}{\cal T}v_gd$. Here, ${\cal T}$ is the average time between consecutive encounters of the clock with DM objects, which, for a given *ρ*_DM_, depends on the energy density inside the defect^6^ $\rho _{{\mathrm{inside}}} = \rho _{{\mathrm{DM}}}{\cal T}v_g/d.$ Thus the expected DM-induced fractional frequency excursion reads

4 $$\frac{{\delta \omega _{{\mathrm{ext}}}}}{{\omega _c}} = {\it{\Gamma }}_{{\mathrm{eff}}}\hbar c\rho _{{\mathrm{DM}}}v_g{\cal T}d,$$

which is valid for TDs of any type (monopoles, walls and strings). The frequency excursion is positive for *Γ*_eff_ > 0, and negative for *Γ*_eff_ < 0.

The key qualifier for the preceding Eq. (4) is that one must be able to distinguish between the clock noise and DM-induced frequency excursions. Discriminating between the two sources relies on measuring time delays between DM events at network nodes. Indeed, if we consider a pair of spatially separated clocks (Fig. 2), the DM-induced frequency shift Eq. (2) translates into a distinct pattern. The velocity of the sweep is encoded in the time delay between two DM-induced spikes and it must lie within the boundaries predicted by the standard halo model. Generalisation to the multi-node network is apparent (see GPS-specific discussion below). The distributed response of the network encodes the spatial structure and kinematics of the DM object, and its coupling to atomic clocks.

### Analysis and search

Working with GPS data introduces several peculiarities into the above discussion (see Supplementary Note 1 for details). The most relevant is that the available GPS clock data are clock biases (i.e., time differences between the satellite and reference clocks) *S* ^(0)^(*t*_*k*_) sampled at times (epochs) *t*_*k*_ every 30 s. Thus we cannot access the continuously sampled clock frequencies as in Fig. 2. Instead, we formed discretised pseudo-frequencies $S^{(1)}(t_k) \equiv S^{(0)}(t_k) - S^{(0)}(t_{k - 1})$. Then the signal is especially simple if the DM object transit time through a given clock, *d*/*v*_*g*_, is smaller than the 30-s epoch interval (i.e., thin DM objects with $d \lesssim 10^4\,{\mathrm{km}}$, roughly the size of the Earth), since in this case *S* ^(1)^ collapses into a solitary spike at *t*_*k*_ if the DM object was encountered during the (*t*_*k*−1_, *t*_*k*_) interval. The exact time of interaction within this interval is treated as a free parameter.

**Fig. 3:**
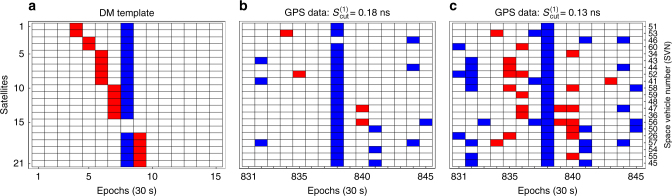
Correlated dark matter signal across satellite network. **a** One of the expected pseudo-frequency *S* ^(1)^ signatures for a thin domain wall. Red (blue) tiles indicate positive (negative) dark matter-induced frequency excursions, while white tiles mark the absence of the signal (c.f. Fig. 2). In this example, the satellites are listed in the order they were swept (though in general the order depends on the incident direction of the dark matter object and is not known a priori), and *Γ*_eff_ > 0 in Eq. (1). The slope of the red line encodes the incident velocity of the wall. The reference clock was swept within the 30 s leading to epoch 8. Satellites 15 and 16 do not record any frequency excursions, since they are spatially close the reference clock and are swept within the same 30 s period. **b**, **c** Show *S* ^(1)^ atomic clock data streams for all operational Rb Global Positioning System satellite clocks for 21 May, 2010 for a 15 epoch window. Red tiles show data points with $S^{(1)}  >S_{{\mathrm{cut}}}^{(1)}$, and the blue depict $S^{(1)} < - S_{{\mathrm{cut}}}^{(1)}$, with $S_{{\mathrm{cut}}}^{(1)} = 0.18\,{\mathrm{ns}}$ and 0.13 ns, respectively. At the 0.13 ns level, **c**, this data window would be flagged as a potential event, but not at the 0.18 ns level shown in **b**. In this case, the potential event **c** is excluded because the reference clock experiences a much larger perturbation than the rest of the clock network

One of the expected *S* ^(1)^ signatures for a thin domain wall propagating through the GPS constellation is shown in Fig. 3a. This signature was generated for a domain wall incident with *v* = 300 km s^−1^ from the most probable direction. The derivation of the specific expected domain wall signal is presented in Supplementary Note 3, which includes Supplementary Fig. 3. As the DM response of Rb and Cs satellite clocks can differ due to their distinct effective coupling constants *Γ*_eff_, we treated the Cs and Rb satellites as two sub-networks, and performed the analysis separately. Within each sub-network we chose the clock on board the most recently launched satellite as the reference because, as a rule, such clocks are the least noisy among all the clocks in orbit.

To search for domain wall signals, we analysed the *S* ^(1)^ GPS data streams in two stages. At the first stage, we scanned all the data from May 2000 to October 2016 searching for the most general patterns associated with a domain wall crossing, without taking into account the order in which the satellites were swept. We required at least 60% of the clocks to experience a frequency excursion at the same epoch, which would correspond to when the wall crossed the reference clock (vertical blue line in Fig. 3a). This 60% requirement is a conservative choice based on the GPS constellation geometry, and ensures sensitivity to walls with relative speeds of up to $v \lesssim 700\,{\mathrm{km}}\,{\kern 1pt} {\mathrm{s}}^{ - 1}$. Then, we checked if these clocks also exhibit a frequency excursion of similar magnitude (accounting for clock noise) and opposite sign anywhere else within a given time window (red tiles in Fig. 3a). Any epoch for which these criteria were met was counted as a potential event. We considered time windows corresponding to sweep durations through the GPS constellation of up to 15,000 s, which is sufficiently long to ensure sensitivity to walls moving at relative velocities $v \,\lesssim\, 4\,{\mathrm{km}}\,{\kern 1pt} {\mathrm{s}}^{ - 1}$ (given that <0.1% of DM objects are expected to move with velocities outside of this range). Further details of the employed search technique are presented in the Methods section and Supplementary Note 4.

The tiled representation of the GPS data stream depends on the chosen signal cut-off $S_{{\mathrm{cut}}}^{(1)}$ (see Fig. 3). We systematically decreased the cut-off values and repeated the above procedure. Above a certain threshold, $S_{{\mathrm{thresh}}}^{(1)}$, no potential events were seen. This process is demonstrated for a single arbitrarily chosen data window in Fig. 3b, c. The thresholds for the Rb and Cs subnetworks above which no potential events were seen are $S_{{\mathrm{thresh}}}^{(1)}\left( {{\mathrm{Rb}}} \right) = 0.48\,{\mathrm{ns}}$ and $S_{{\mathrm{thresh}}}^{(1)}({\mathrm{Cs}}) = 0.56\,{\mathrm{ns}}$ for *v *≈ 300 km s^−1^ sweeps.

The second stage of the search involved analysing the potential events in more detail, so that we may elevate their status to candidate events if warranted by the evidence. We examined a few hundred potential events that had *S* ^(1)^ magnitudes just below $S_{{\mathrm{thresh}}}^{(1)}$, by matching the data streams against the expected patterns; one such example is shown in Fig. 3a. At this second stage, we accounted for the ordering and time at which each satellite clock was affected. The velocity vector and wall orientation were treated as free parameters within the bounds of the standard halo model. As a result of this pattern matching, we found that none of these events were consistent with domain wall DM, thus we have found no candidate events at our current sensitivity. Analysing numerous potential events well below $S_{{\mathrm{thresh}}}^{(1)}$ has proven to be substantially more computationally demanding, and is beyond the scope of the current work.

## Discussion

As we did not find evidence for encounters with domain walls at our current sensitivity, there are two possibilities: either DM of this nature does not exist, or the DM signals are below our sensitivity. In the latter case we may constrain the possible range of the coupling strengths *Γ*_eff_. For the discrete pseudo-frequencies, and considering the case of thin domain walls, Eq. (4) becomes

5 $$\left| {{\it{\Gamma }}_{{\mathrm{eff}}}} \right| < \frac{{S_{{\mathrm{thresh}}}^{(1)}}}{{\hbar c\sqrt \pi \rho _{{\mathrm{DM}}}\,{\cal T}\,s(d){\kern 1pt} d^2}}.$$

Our technique is not equally sensitive to all values for the wall widths, *d*, or average times between collisions, ${\cal T}$. This is directly taken into account by introducing a sensitivity function, *s*(*d*)∈[0,1], that is included in Eq. (5) to determine the final limits at the 90% confidence level. For example, the smallest width is determined by the servo-loop time of the GPS clocks, i.e., by how quickly the clock responds to the changes in atomic frequencies. In addition, we are sensitive to events that occur less frequently than once every ~ 150 s (so the expected patterns do not overlap), which places the lower bound on ${\cal T}$. Further, we incorporate the expected event statistics into Eq. (5). Details are presented in the Methods section.

**Fig. 4:**
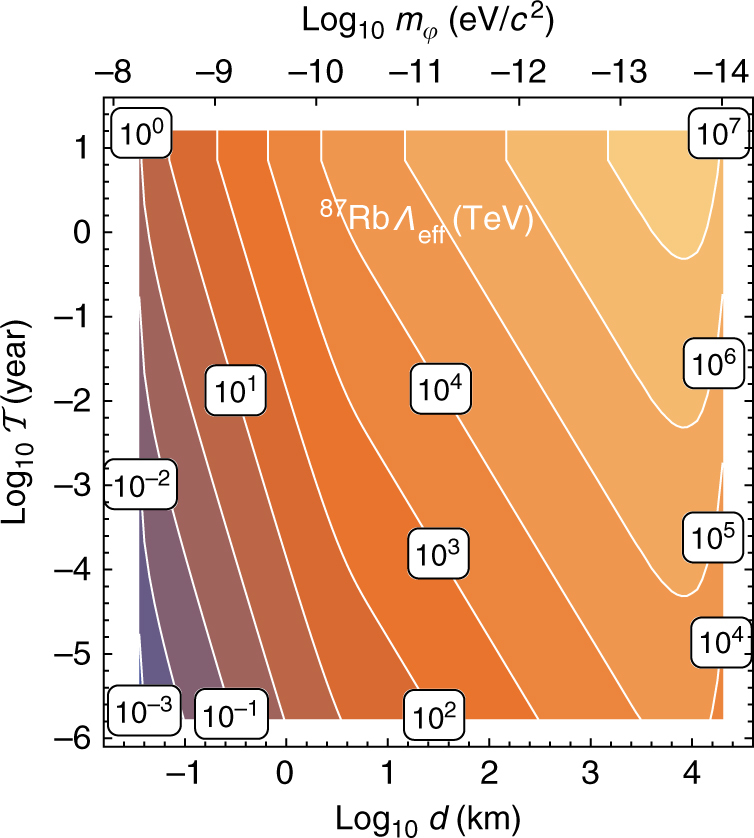
Results for the effective energy scale. Contour plot showing the 90% confidence level exclusion limits on the effective energy scale *Λ*_eff_ from the Global Positioning System Rb sub-network as a function of the wall width, *d*, and average time between encounters with domain walls, ${\cal T}$. The secondary horizontal axis shows the dark matter field mass, which for topological defects is related to the width via $m_\varphi \sim \hbar /dc$

**Fig. 5:**
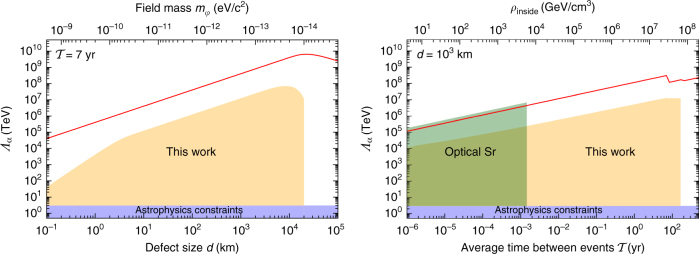
Constraints on the coupling of dark matter to electromagnetism. Limits (90% confidence level) on the energy scale *Λ*_*α*_ as a function of the wall width *d* and average time between encounters ${\cal T}$. The shaded yellow region shows the Global Positioning System limits from this work (assuming ${\it{\Gamma }}_\alpha \gg {\it{\Gamma }}_{q,e/p}$), the shaded green region shows the limits derived from an optical Sr clock^38^, and the shaded blue region shows the astrophysical bounds^39^. The solid red line shows the potential discovery reach using the global network of Global Positioning System microwave atomic clocks. For ${\cal T} \lesssim 7\,{\mathrm{yr}}$, the Global Positioning System reach is limited by the modern Rb block IIF satellite clocks^46^ ($\sigma _y(30\,{\mathrm{s}})\sim 10^{ - 11}$), and for ${\cal T} \lesssim 7\,{\mathrm{yr}}$, the reach is limited by the older Rb (block IIR, IIA and II) clocks ($\sigma _y(30\,{\mathrm{s}})\sim 10^{ - 10}$). Compared to more accurate optical clocks, microwave clocks provide additional sensitivity to *Λ*_*q*_ and *Λ*_*e*/*p*_ (optical clocks only have sensitivity to *Λ*_*α*_)

Our results are presented in Fig. 4. To be consistent with previous literature^6,38^, the limits are presented for the effective energy scale $\Lambda _{{\mathrm{eff}}} \equiv 1/\sqrt {\left| {{\it{\Gamma }}_{{\mathrm{eff}}}} \right|}$. Further, on the assumption that the coupling strength *Γ*_*α*_ dominates over the other couplings in the linear combination in Eq. (3), we place limits on *Λ*_*α*_. The resulting limits are shown in Fig. 5, together with existing constraints^38,39^. For certain parameters, our limits exceed the 10^7^ TeV level; astrophysical limits^39^ on *Λ*_*α*_, which come from stellar and supernova energy-loss observations^40,41^, have not exceeded ~ 10 TeV.

The derived constraints on *Λ*_*α*_ can be translated into a limit on the transient variation of the fine-structure constant,

6 $$\frac{{\delta \alpha }}{\alpha } = \hbar c\rho _{{\mathrm{DM}}}v_g\frac{{{\cal T}d}}{{\Lambda _\alpha ^2K_\alpha }},$$

which for *d* = 10^4^ km corresponds to $\delta \alpha /\alpha \lesssim 10^{ - 12}$. Because of the scaling of the constraints on *Λ*_*X*_, this result is independent of ${\cal T}$, and scales inversely with *d* (within the region of applicability). It is worth contrasting this constraint with results from the searches for slow linear drifts of fundamental constants. For example, the search^5^ resulting in the most stringent limits on long-term drifts of *α* was carried out over a year and led to $\frac{{\delta \alpha }}{\alpha } \lesssim 3 \times 10^{ - 17}$. Such long-term limits apply only for very thick walls of thickness $d \gg v_g \times 1\,{\mathrm{yr}}\sim 10^{10}\,{\mathrm{km}}$, which are outside our present discovery reach.

**Fig. 6:**
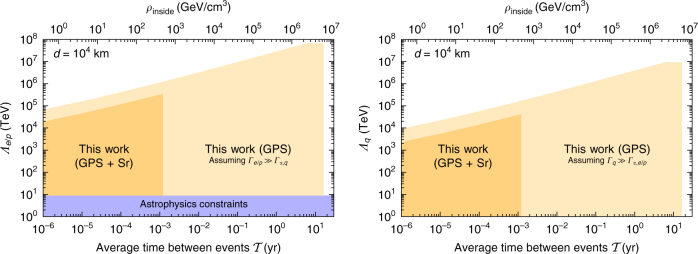
Constraints on the coupling of dark matter to fermion masses. Limits (90% confidence level) on the energy scales *Λ*_*e*/*p*_ and *Λ*_*q*_ as a function of the average time between encounters, ${\cal T}$, for constant *d *= 10^4^ km. The lighter yellow regions are the limits from the Rb Global Positioning System sub-network in the assumption that the respective couplings dominate the interactions. The darker region combines our limits (from the Rb and Cs sub-networks) with the limits on *Λ*_*α*_ from the Sr optical clock constraints^38^ to place assumption-free limits on *Λ*_*e*/*p*_ and *Λ*_*q*_. The blue region shows the astrophysical bounds for *Λ*_*e*/*p*_; note that *Λ*_*q*_ was previously unconstrained^39^

Further, by combining our results from the Rb and Cs GPS sub-networks with the recent limits on *Λ*_*α*_ from an optical Sr clock^38^, we also place independent limits on *Λ*_*e*/*p*_, and *Λ*_*q*_; for details, see Supplementary Note 5. These limits are presented in Fig. 6 as a function of the average time between events. For certain values of the *d* and ${\cal T}$ parameters, we improve current bounds on *Λ*_*e*/*p*_ by a factor of ~ 10^5^ and for the first time establish limits on *Λ*_*q*_.

While we have improved the current constraints on DM-induced transient variation of fundamental constants by several orders of magnitude, it is possible that DM events remain undiscovered in the data noise. Our current threshold $S_{{\mathrm{thresh}}}^{(1)}$ is larger than the GPS data noise by a factor of ~ 5–20, depending on which clocks/time periods are examined. By applying a more sophisticated statistical approach with greater computing power, we expect to improve our sensitivity by up to two orders of magnitude. Indeed, the sensitivity of the search is statistically determined by the number of clocks in the network, *N*_clocks_, and the Allan deviation^42^, *σ*_*y*_(*τ*_0_), evaluated at the data sampling interval *τ*_0_ = 30 s reads,

7 $$S^{(1)} \lesssim \frac{{\sigma _y\left( {\tau _0} \right)\,\tau _0}}{{\sqrt {N_{{\mathrm{clocks}}}} }},$$

or, combining with Eq. (5),

8 $$\Lambda _X \lesssim d\sqrt {\frac{{\hbar c\rho _{{\mathrm{DM}}}{\cal T}\,K_X\sqrt {N_{{\mathrm{clocks}}}} }}{{\sigma _y\left( {\tau _0} \right)\,\tau _0}}} .$$

Note that this estimate differs from a previous estimate^6^, since while arriving at Eq. (7), we assumed a more realistic white frequency noise (instead of white phase noise). The projected discovery reach of GPS data analysis is presented in Fig. 5.

Prospects for the future include incorporating substantially improved clocks on next-generation satellites, increasing the network density with other Global Navigation Satellite Systems, such as European Galileo, Russian Global Navigation Satellite System (GLONASS), and Chinese BeiDou, and including networks of laboratory clocks^38,43^. Such an expansion can bring the total number of clocks to ~ 100. Moreover, the GPS search can be extended to other TD types (monopoles and strings), as well as different DM models, such as virialized DM fields^32,44^.

In summary, by using the GPS as a dark matter detector, we have substantially improved the current limits on DM domain wall induced transient variation of fundamental constants. Our approach relies on mining an extensive set of archival data, using existing infrastructure. As the direct DM searches are widening to include alternative DM candidates, it is anticipated that the mining of time-stamped archival data, especially from laboratory precision measurements, will play an important role in verifying or excluding predictions of various DM models^45^. In the future, our approach can be used for a DM search with nascent networks of laboratory atomic clocks that are orders of magnitude more accurate than the GPS clocks^43^.

## Methods

### Crossing duration distribution

Before placing limits on *Γ*_eff_, we must account for the fact that we do not have equal sensitivity to each domain wall width, *d*, or equivalently crossing durations,

9 $$\tau \equiv \frac{d}{{v_ \bot }},$$

where *v*_⊥_ is the component of the velocity perpendicular to the face of the DM wall. This is due in part to aspects of the clock hardware and operation, the time-resolution (data sampling frequency), and the employed search method. Therefore, for a given *d*, we must determine the proportion of events that have crossing durations within the range *τ*_min_ to *τ*_max_, where *τ*_min(max)_ is the minimum (maximum) crossing duration for which this method is sensitive.

There are two factors that determine *τ*_min_. The first is the servo-loop time—the fastest perturbation that can be recorded by the clock. This servo-loop time is manually adjusted by military operators and is not available to us at a given epoch, however, it is known^46,47^ to be within 0.01 and 0.1 s. As such, we consider the best and worse case scenarios:

10 $$\begin{array}{*{20}{l}} {\tau _{{\mathrm{min}}}^{{\mathrm{Servo}}}\left( {{\mathrm{best}}} \right) = 0.01\,{\mathrm{s}},} \hfill \\ {\tau _{{\mathrm{min}}}^{{\mathrm{Servo}}}\left( {{\mathrm{worst}}} \right) = 0.1\,{\mathrm{s}}.} \hfill \end{array}$$

Note, that below *τ* ^Servo^ we still have sensitivity to DM events, however, the sensitivity in this region is determined by the response of the quartz oscillator to the temporary variation in fundamental constants. However, the resulting limits for crossing durations shorter than the servo-loop time are generally weaker than the existing astrophysics limits (see Supplementary Note 5, including Supplementary Figs. 4–8), so we consider this no further.

The second condition that affects *τ*_min_ is the clock degeneracy: the employed GPS data set has only 30 s resolution, so any clocks which are affected within 30 s of the reference clock will not exhibit any DM-induced frequency excursion in their data; see satellites 15 and 16 in Fig. 3a. For <60% of the clocks to experience the jump, the (thin-wall) DM object would have to be travelling at over 700 km s^−1^, which is close to the galactic escape velocity (for head-on collisions), so the degeneracy does not affect the derived limits in a substantial way. (In fact, assuming the standard halo model, <0.1% of events are expected to have *v*_⊥_ > 700 km s^−1^.) This velocity corresponds to a crossing duration for the entire network of ~ 70 s. Transforming this to crossing duration for a single clock, *τ*, amounts to multiplying by the ratio *d*/(*D*_G*PS*_):

11 $$\tau _{{\mathrm{min}}}^{{\mathrm{Degen}}.} = \frac{d}{{D_{{\mathrm{GPS}}}}}\,70\,{\mathrm{s}}.$$

For the thickest walls we consider (~ 10^4^ km), this leads to $\tau _{{\mathrm{min}}}^{{\mathrm{Degen}}{\mathrm{.}}}$ of 14 s.

Combining the servo-loop and degeneracy considerations, we arrive at the expression

12 $$\begin{array}{*{20}{l}} {\tau _{{\mathrm{min}}}^{{\mathrm{best}}} = {\mathrm{max}}\left( {0.01{\mathrm{s}}{\kern 1pt} ,\,\frac{d}{{D_{{\mathrm{GPS}}}}}\,70{\kern 1pt} {\mathrm{s}}} \right),} \hfill \\ {\tau _{{\mathrm{min}}}^{{\mathrm{worst}}} = {\mathrm{max}}\left( {0.1{\kern 1pt} {\mathrm{s}}{\kern 1pt} ,{\kern 1pt} \frac{d}{{D_{{\mathrm{GPS}}}}}{\kern 1pt} 70\,{\mathrm{s}}} \right).} \hfill \end{array}$$

For walls thicker than *d* ~ 100 km, *τ*_min_ is determined by the *d*/(*D*_GPS_)70 s term.

As to the maximum crossing duration, there are also two factors that affect *τ*_max_. First, the wall must pass each clock in less than the sampling interval of 30 s—this is the condition for the wall to be considered thin:

13 $$\tau _{{\mathrm{max}}}^{{\mathrm{thin}}} = 30\,{\mathrm{s}}.$$

If a wall takes longer than 30 s to pass by a clock, the simple single-data–point signals shown in Fig. 3 would become more complicated, and would require a more-detailed pattern-matching technique. Second, we only consider time windows, *J*_*w*_, of a certain size in our analysis (see Supplementary Note 4). If a wall moves so slowly that it does not sweep all the clocks within this window, the event would be missed:

14 $$\tau _{{\mathrm{max}}}^{{\mathrm{window}}} = J_w\frac{d}{{D_{{\mathrm{GPS}}}}}.$$

Therefore, the overall expression for *τ*_max_ is:

15 $$\tau _{{\mathrm{max}}} = {\mathrm{min}}\left( {30\,{\mathrm{s}},\,J_w\frac{d}{{D_{{\mathrm{GPS}}}}}} \right).$$

Making *J*_*w*_ large, however, also tends to increase $S_{{\mathrm{thresh}}}^{(1)}$ (since there is a higher chance that a large window will satisfy the condition for a potential event). By performing the analysis for multiple values for *J*_*w*_, we can probe the largest portion of the parameter space; for further details see Supplementary Note 4 and Supplementary Tables 2 and 3. In this work, we consider windows of *J*_*w*_ up to 500 epochs (15,000 s), which corresponds to a minimum velocity of ~ 4 km s^−1^, which is roughly the orbit speed of the satellites. This has a negligible effect on our sensitivity, since <0.1% of walls are expected to have *v*_⊥_ < 4 km s^−1^.

### Domain wall width sensitivity

Assuming the standard halo model, the relative scalar velocity distribution of DM objects that cross the GPS network is quasi-Maxwellian

16 $$f_v\left( v \right) = \frac{{Cv^2}}{{v_c^3}}\left[ {\exp\left( {\frac{{ - \left( {v - v_c} \right)^2}}{{v_c^2}}} \right) - \exp\left( {\frac{{ - \left( {v + v_c} \right)^2}}{{v_c^2}}} \right)} \right],$$

where *v*_*c*_ = 220 km s^−1^ is the Sun’s velocity in the halo frame, and *C* is a normalisation constant. The form of Eq. (16) is a consequence of the motion of the reference frame. However, the distribution of interest for domain walls is the perpendicular velocity distribution for walls that cross the network

17 $$f_ \bot \left( {v_ \bot } \right) = C{\prime}\mathop {\int}\limits_{v_ \bot }^\infty {f_v(v)\frac{{v_ \bot }}{{v^2}}} \,dv,$$

where *v*_⊥_ is the component of the wall’s velocity that is perpendicular to the wall, and *C*′ is a normalisation constant. Note that this is not the distribution of perpendicular velocities in the galaxy—instead, it is the distribution of perpendicular velocities that are expected to cross paths with the GPS constellation (walls with velocities close to parallel to face of the wall are less likely to encounter the GPS satellites, and objects with higher velocities more likely to).

Now, define a function *f*_*τ*_(*d*, *τ*), such that the integral ${\int}_{\tau _a}^{\tau _b} {f_\tau (d,\tau )d\tau }$ gives the fraction of events due to walls of width *d* that have crossing durations between *τ*_*a*_ and *τ*_*b*_. Note, this function must have the following normalisation:

$$\mathop {\int}\limits_0^\infty {f_\tau \left( {d,\tau } \right)} \,{\mathrm{d}}\tau = 1$$

for all *d*, and is given by

18 $$f_\tau \left( {d,\tau } \right) = \frac{d}{{\tau ^2}}f_ \bot \left( {v_ \bot } \right).$$

**Fig. 7:**
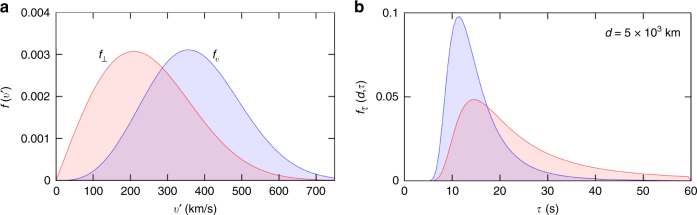
Velocity and crossing time distributions. **a** The blue curve shows the velocity distribution for dark matter objects that cross the Global Positioning System constellation Eq. (16) while the red curve shows the corresponding distribution for the velocity component normal to the domain wall Eq. (17). **b** The resultant single-clock crossing-time, *τ* = *d*/*v*′, distribution for walls of width *d* = 5 × 10^3^ km

Plots of the velocity and crossing-time distributions are given in Fig. 7. Then, our sensitivity at a particular wall width is

19 $$s\left( d \right) = \mathop {\int}\limits_{\tau _{{\mathrm{min}}}}^{\tau _{{\mathrm{max}}}} {f_\tau \left( {d,\tau } \right)} \,d\tau .$$

**Fig. 8:**
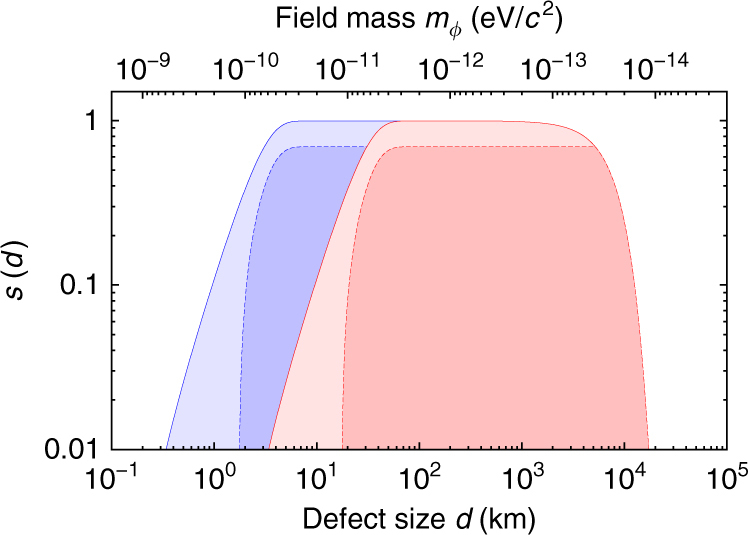
Domain wall width sensitivity. Sensitivity, *s*, as a function of the wall width *d*. The blue region corresponds to the best case scenario (due to the servo-loop uncertainty), and the red region to the worst case, see Eq. (12). The darker regions correspond to a time window of size *J*_*w*_ = 300 s (i.e., *v*_min_ ≈ 170 km s^−1^), and the lighter regions correspond to *J*_*w*_ = 3000 s (*v*_min_ ≈ 17 km s^−1^). The *upper* horizontal axis shows the mass of the underlying dark matter field

Plots of the sensitivity function for a few various cases of parameters are presented in Fig. 8.

### Data availability

We used publicly available GPS timing and orbit data for the past 16 years from the Jet Propulsion Laboratory^10^.

## Electronic supplementary material

Supplementary Information
