# Supplementary material for: Search for domain wall dark matter with atomic clocks on board global positioning system satellites
Source: Nat Commun. 2017 Oct 30;8:1195. doi: 10.1038/s41467-017-01440-4 (PMC5662606; doi:10.1038/s41467-017-01440-4)
Supplement: Supplementary file 1 — Supplementary Information [file 41467_2017_1440_MOESM1_ESM.pdf]

Supplementary Note 1

GPS data processing and clock estimation

The Global Positioning System (GPS) works by broadcasting microwave signals from nominally 32 satellites in medium-Earth orbit. The signals are driven by an atomic clock (either based on Rb or Cs atoms) on board each satellite. While each satellite may host multiple Rb and Cs backup clocks, at any given time only one of these clocks drives the GPS signals, which are transmitted on carrier waves at both L1 ($1.57542\,\mathrm{GHz}$) and L2 ($1.2276\,\mathrm{GHz}$) bands. Superimposed on the carrier waves are streams of pseudo-random bits generated by flipping the sign of the electric field. A geodetic GPS receiver can sample the dual-frequency signals simultaneously from all GPS satellites in view (typically 8 to 10) at user-specified intervals (typically 1 to 30 seconds). At every such interval, for each satellite in view, timing measurements are made (according to the receiver clock) of the peak cross-correlation of the incoming signal with the receiver's replica model of the signal. At both L1 and L2 frequencies, two types of data are generated including a pseudorange (group delay, using the bits) and a carrier phase (phase delay, using the carrier wave). The term pseudorange is used because it is a measure of delay that is biased by the receiver clock. This bias cancels when differencing data between pairs of satellites. The ionospheric delay of $\sim 10\,\mathrm{ns}$ is calibrated with $\sim 0.02\,\mathrm{ns}$ precision by forming a specific ionosphere-free linear combination of the data at L1 and L2 frequencies, hence the purpose of the dual-frequency system.

Measurement precision in such cross-correlation systems tends to scale with the relevant wavelength of the signal. In the case of pseudorange, precision scales with the time interval between bit transitions. As a consequence, the pseudorange precision is typically $\sim 2\,\mathrm{ns}$, whereas in contrast, carrier phase is measured with $\sim 0.02\,\mathrm{ns}$ precision, but suffers from a constant integer cycle bias that is initially unknown. Resolving this bias is known as integer ambiguity resolution. Combinations of the 4 observables (pseudorange and carrier phase on both frequencies) allow for robust detection of data outliers and cycle slips in the integer bias, and enable robust integer ambiguity resolution[1,2]. With integer ambiguities resolved, carrier phase data can then be modelled as pseudorange data, but with two orders of magnitude more precision.

Here we analyse data from the Jet Propulsion Laboratory (JPL)[3], in which the clock biases are given at $30\,\mathrm{s}$ intervals[4]. These clock biases are generated using data from a global network of ~ 100 GPS geodetic receivers by a mature analysis system that is used routinely for purposes of centimetre-level satellite orbit determination, and millimetre-level positioning for scientific purposes, such as plate tectonics, Earth rotation, and geodynamics. The analysis standards that are applied are consistent with models and conventions specified by the International Earth Rotation and Reference Frames Service (IERS), in line with resolutions of the International Astronomical Union (IAU). These standards ensure best practices and consistency between various geodetic techniques, including very long baseline interferometry (VLBI), and satellite laser ranging (SLR), both of which have been instrumental in developing the IERS conventions. We note that similar types of analyses abiding by the IERS conventions are performed by several analysis centres around the world, and are routinely compared by the International Global Navigation Satellite System Service (IGS). Results of these comparisons, along with the variety of scientific applications that depend on such data, provide an abundance of evidence that the clock biases are determined relative to each other with an accuracy $< 0.1\,\mathrm{ns}$.

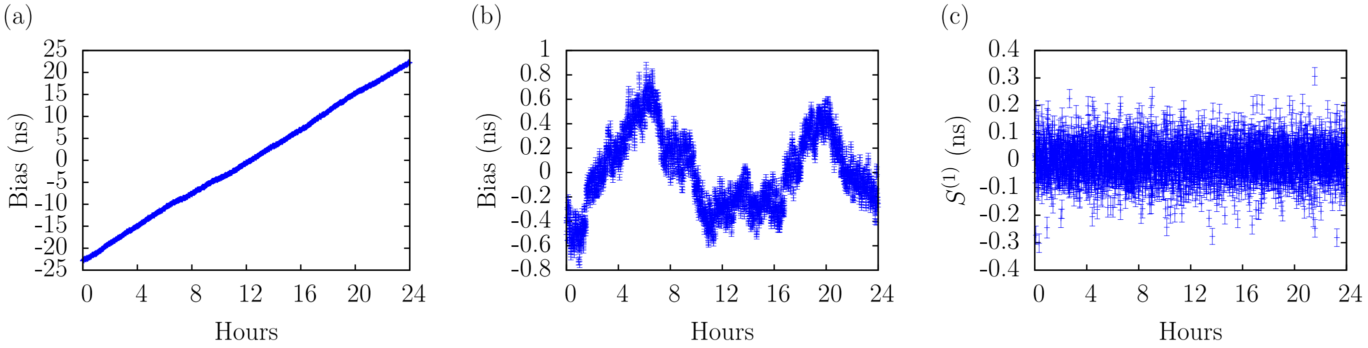

Supplementary Figure 1: Plots of clock data from an arbitrarily chosen Rb clock. Block IIR, Space Vehicle Number 61, see Ref. 5, for 27 December 2015 UTC; the USN7 H-maser receiver-station clock 6 was used as reference. (a) Raw clock bias, with a constant offset removed. (b) With a second-order polynomial fit and subtracted to reveal the sub-daily variance. (c) First-order differenced $S^{(1)}$ data. The error bars shown in the plots are the formal errors. Note that the differenced data $S^{(1)}$ is generated directly from raw clock bias (no fit polynomial is removed).

Analysis of GPS receiver data includes the effects of special and general relativity. All ideal clocks stationary on one of the Earth's equipotential surfaces such as the geoid (sea level) have no relative phase drift. For purposes of discussion, let us define coordinate time as proper time on the geoid. (Actually a different coordinate time is chosen in geodesy, but that does not change the argument here.) For each satellite, the combination of velocity and gravitational potential causes satellite proper time to vary with respect to coordinate time. This is dominated by a positive drift at the level of 0.45 parts per billion, which would be $\sim 13\,\mathrm{ns}$ per $30\,\mathrm{s}$ epoch. The hardware in the GPS satellite is designed to set the effective frequency of the atomic clocks such that the drift is zero on average, using the known semi-major axis of each satellite's orbit. A residual effect results from the satellite moving in an ellipse, and thus with a time varying velocity and gravitational potential. The eccentricity of the GPS satellites is typically small $\lesssim 0.02$; nevertheless, the effect is a periodic variation of satellite proper time with an amplitude of $\sim 30\,\mathrm{ns}$ at $\sim 12\,\mathrm{hr}$ orbit period. This is modelled to first order by knowing the satellite's position and velocity, and by considering the Earth as a spherically symmetric mass. All higher order effects effectively go into the definition of the satellite clock bias provided by JPL, consistent with international conventions. These residual effects are less than $1\,\mathrm{ns}$ over the $\sim 12\,\mathrm{hr}$ orbit period, and so are completely negligible over the time periods investigated here.

In JPL's global GPS data analysis, the results of which are employed as input data to our dark matter (DM) search, the clock biases are estimated using GPS ionosphere-free carrier phase and pseudorange data combinations, as part of a multi-parameter least-squares estimation process using a square-root information filter. Other parameters that are estimated along with the clock biases include GPS orbits, station positions, Earth rotation, and atmospheric delay. Correlated errors between the clocks and other parameters are formally computed by the square-root information filter to be at the level $< 0.1\,\mathrm{ns}$, consistent with the inferred level of accuracy.

In this process, there is effectively no restriction on the allowed behaviour of the clocks from one $30\,\mathrm{s}$ epoch to the next. Crucially, if a clock were to have a real transient that far exceeded engineering expectations, the data over that time window would not have been removed as outliers. Since only relative clock bias can be estimated, one clock is always held fixed in the estimation procedure. The choice of reference clock is irrelevant, in that our search algorithms look at differences in estimated clock biases.

In order to analyse the estimated clock biases, we further apply a first-order differencing procedure to the data, and define the pseudo-frequencies

$$S^{(1)}(t_k) = S^{(0)}(t_k) - S^{(0)}(t_{k-1}), \tag{1}$$

where $S^{(0)}(t_k)$ is the original clock bias for the t_k epoch (data point), and $t_k - t_{k-1} = 30\,\mathrm{s}$ is the sampling

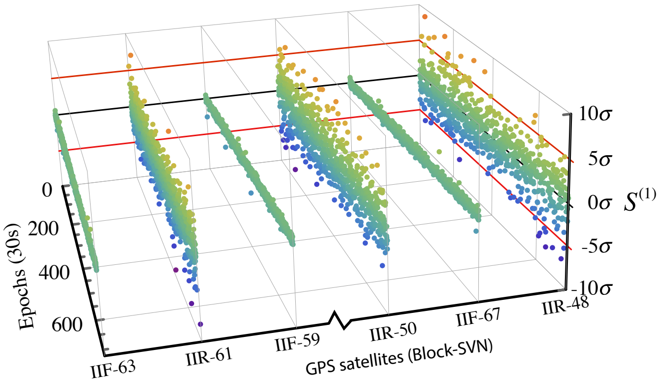

Supplementary Figure 2: The first 6 hours of single-differenced clock data $S^{(1)}$, in units of the formal error σ, for 5 June 2016 (UTC), for six arbitrarily chosen Rb clocks. The clocks are labelled by their satellite block (either II, IIA, IIR or IIF), and their Space Vehicle Number (SVN); see Ref. 5.

Supplementary Table 1: Typical standard deviations (in ns) of the $S^{(1)}$ clock data over the 16 years analysed in this work. The standard deviations vary significantly between the satellite generations[5] (II, IIA, IIR, and IIF), and also depend on whether the ground based H-maser clock was used as reference (in this case USN3[6]), or one of the other Global Positioning System satellite clocks.

Generation	Reference	
	USN3	RbIIR
Rb-II	0.047	0.070
Rb-IIA	0.038	0.074
Rb-IIR	0.073	0.097
Rb-IIF	0.013	0.067
Generation	Reference	
	USN3	CsIIA
Cs-II	0.081	0.112
Cs-IIA	0.088	0.124
Cs-IIF	0.098	0.128

interval. This is equivalent to taking a discrete derivative (up to a multiplicative factor), and acts to whiten the data (since the clock bias noise is dominated by random walk processes). This differencing procedure also removes any constant bias offsets, and transforms the linear frequency drifts into constant offsets in $S^{(1)}$. In practice, these residual offsets are small, and are removed by subtracting the mean of $S^{(1)}$ over the given day. The effect of this procedure is demonstrated for a single arbitrarily-chosen clock in Supplementary Figure 1.

The clock biases from JPL[3] also come with a formal error. The formal error quantifies uncertainty in the determination of the clock bias, and does not directly incorporate the intrinsic clock noise. It varies slowly over time, and is typically on the order of $\sim 0.02 - 0.03\,\mathrm{ns}$; the formal error is dominated by the uncertainty in the satellite orbit determination. Only the most recent satellite clocks have observed temporal variations in $S^{(1)}$ that are at a similar level as the formal error, indicating that temporal variations in older clocks are actually due to clock behaviour rather than estimation error. A snapshot of the standard deviations for the $S^{(1)}$ clock data used in our analysis is presented in Supplementary Table 1. A plot showing a few hours of $S^{(1)}$ data for an arbitrarily selected few satellite clocks is shown in Supplementary Figure 2. This shows quite clearly how the more modern block IIF satellites[5] are substantially less noisy than the older-generations of satellites (by orders of magnitude).

Since the clocks are noisy, in order to discern the DM-induced signal from the intrinsic clock noise we rely on signals correlated across the entire GPS network, as discussed in the following sections. Moreover,

there already exists more than 15 years of high-accuracy timing data that can be exploited in the search. This data stream is being routinely updated, and, in principal, timing data from any other atomic clocks that are synchronised with GPS can be included in the analysis (for a discussion on this point, see Ref. 7). Therefore, by analysing the new and existing GPS timing data, we can perform a sensitive search for transient DM signals, and if no signals are found we can then place limits on the DM–ordinary matter interaction strengths[8]. The GPS network is particularly well suited for this type of search, for a number of reasons. The large number of clocks, and the very large ($\sim 50\,000\,\mathrm{km}$) diameter of the network increases both the chance of an interaction, and the sensitivity of the search, since we rely on correlated signal throughout the entire network. Similar arguments underpin motivations for searches using global network of atomic magnetometers[9,10]. Such magnetometry searches are sensitive to different types of interactions (as discussed below), and are therefore complementary to atomic clock searches.

Supplementary Note 2

Using GPS to search for topological defects

The null results from recent weakly-interacting massive particle (WIMP) direct-detection experiments have partly contributed to increased attention to ultralight field DM, such as axions[11–15]. Ultralight fields may form stable topological defects (TDs), such as monopoles, strings, or domain walls, which can be a dominant or subdominant contribution to both DM and dark energy[16–22]. The interactions of light fields with standard model (SM) fields can be written as a sum of effective interaction Lagrangians (so-called 'portals')[8]

$$\mathcal{L}_{\text{int}} = \mathcal{L}^{\mathrm{PS}} + \mathcal{L}^{\mathrm{S}^1} + \mathcal{L}^{\mathrm{S}^2} + \ \ldots\ , \tag{2}$$

where $\mathcal{L}^{\mathrm{PS}}$ represents the pseudoscalar (axionic) portal, and $\mathcal{L}^{\mathrm{S}^1}$ and $\mathcal{L}^{\mathrm{S}^2}$ are the linear and quadratic scalar portals, respectively. The linear and quadratic scalar portals, as will be demonstrated below, lead to changes in the effective values of certain fundamental constants and thus cause shifts in atomic transition frequencies, and so lend themselves well to searches based on the use of atomic clocks. The axionic portal leads to interactions that could cause spin-dependent shifts due to fictitious magnetic fields, and are thus are well suited to magnetometry searches[9,10]. We note that there are very stringent limits on the interaction strength for the linear scalar interaction coming from astrophysics and gravitational experiments (see, e.g., Refs. 23,24). However, the constraints on the quadratic portal are substantially weaker[25]. For this reason, we will concentrate on the quadratic scalar portal.

While we mainly address topological defect dark matter, and in particular refer the quadratic scalar coupling, it is important to note that our experiment is not limited in scope to this possibility. Any large (on laboratory scales), clumpy object (e.g., Q-balls[26–28], strings[29], solitons[30–32], and other stable objects[33–35]) that interacts with standard model particles in such a way that leads to shifts in atomic transition frequencies is possible to detect using this scheme.

In the assumption of a quadratic scalar coupling between the standard model (SM) and DM fields, the interaction Lagrangian can be parameterized as[8]

$$-\,\mathcal{L}^{\mathrm{DM-SM}} = \varphi^2\left(\mathbf{r},t\right)\left(\mathit{\Gamma}_f\, m_f c^2 \bar{\psi}_f \psi_f + \frac{\mathit{\Gamma}_\alpha}{4} F_{\mu\nu}F^{\mu\nu} + \ \ldots\right), \tag{3}$$

where φ is the DM field, m_f are the fermion masses, ψ_f and $F_{\mu\nu}$ are the SM fermion fields and electromagnetic field tensor, respectively, and there is an implicit sum over f that runs over all SM fermions. The constants $\mathit{\Gamma}$ quantify the strengths of the various DM–SM couplings. From a comparison with the conventional SM Lagrangian,

$$-\mathcal{L}^{\mathrm{SM}} = m_f c^2\, \bar{\psi}_f \psi_f + \frac{1}{4} F_{\mu\nu}F^{\mu\nu} + \ \ldots\ ,$$

it is seen that (to lowest order) the above Lagrangian (3) leads to the effective redefinition of certain dimensionless combinations of fundamental constants:

$$\alpha^{\text{eff}} = \alpha \left(1 + \Gamma_\alpha \, \varphi^2\right) \ , \tag{4}$$

$$m_{e,p}^{\text{eff}} = m_{e,p} \left(1 + \Gamma_{m_{e,p}} \, \varphi^2\right) \ , \tag{5}$$

$$\left(\frac{m_q}{\Lambda_{\text{QCD}}}\right)^{\text{eff}} = \frac{m_q}{\Lambda_{\text{QCD}}} \left(1 + \Gamma_{m_q/\Lambda_{\text{QCD}}} \, \varphi^2\right) , \tag{6}$$

where α and Λ_{QCD} are the nominal values of the fine structure constant and the QCD energy scale, respectively, and $m_{e,p}$ are the electron and proton masses. In our notation, the DM field has units of energy (E), thereby Γ_X is expressed in units of E^{-2}. Following Ref. 8, and to aid in the comparison with other works, we will present our results in terms of effective energy scales $\Lambda_X = 1/\sqrt{|\Gamma_X|}$ (for $X = \alpha,\, m_e,\, m_p,\, m_q/\Lambda_{\text{QCD}}$). Note that, by the nature of topological defects (TDs), the DM field $\varphi^2 \to 0$ outside the defect, and $\varphi^2 \to \varphi^2_{\max}$ inside the defect; as such the redefined coupling constants are only realised inside the defect. The field amplitude, $\varphi_{\max}$, can be linked to ρ_{inside}, the energy density inside the defect, as

$$\rho_{\text{inside}} = \varphi^2_{\max}/(\hbar c\, d^2), \tag{7}$$

where d is the width of the defect, which is set by the Compton wavelength for the field[8]:

$$d = \hbar/(m_\varphi c). \tag{8}$$

Note the ultra-light mass scale for the fields we are interested in: a roughly Earth-sized defect has a mass scale $m_\varphi \sim 10^{-14}\,\text{eV}$ (m_φ refers to the mass of the field particles, not the mass of the defect itself). We note here that there are other possibilities to search for ultralight DM fields via their non-gravitational interactions; see, e.g., Refs. 9,10,36–45.

From Supplementary Equations (4) – (6), we may relate the DM-induced frequency shift to a transient variation of fundamental constants. The fractional shift in the clock atom transition frequency, ω_c, can be expressed as

$$\frac{\delta\omega(t)}{\omega_c} = \sum_X K_X \frac{\delta X(t)}{X} \equiv \Gamma_{\text{eff}} \, \varphi^2, \tag{9}$$

where K_X are dimensionless sensitivity coefficients, and X runs over fundamental constants from (4)–(5),

$$\frac{\delta X(t)}{X} = \Gamma_X \varphi^2, \tag{10}$$

and the overall effective coupling constant is defined as

$$\Gamma_{\text{eff}} \equiv \sum_X K_X \Gamma_X, \tag{11}$$

which depends on the specific clock transition through the sensitivity coefficients K_X. We also define $\Lambda_{\text{eff}} = 1/\sqrt{|\Gamma_{\text{eff}}|}$, which have a meaning of effective energy scales. The dimensionless sensitivity coefficients K_X are known from atomic and nuclear structure calculations[46]. For example, considering only the variation in the fine structure constant, α, and ignoring relativistic effects, for optical and microwave transitions, the clock frequencies scale as $\omega_c^{\text{opt}} \propto \alpha^2$, and $\omega_c^{\text{mw}} \propto \alpha^4$, respectively. Slight deviations from these dependencies occur due to relativistic corrections to the atomic structure[46,47].

The clocks on board the GPS satellites work by disciplining the frequency of a quartz oscillator to the resonant frequency of the chosen atomic transition; the quartz oscillator drives the GPS L1 and L2 microwave signals[48]. In order to make a meaningful analysis of the data we must analyse the comparison of the output

frequencies to the frequency of another reference clock. For thin domain walls, the GPS clock and the reference clock are spatially separated, so only one is affected by the TD at a given time. (When both clocks are affected at the same time we would see no DM signal, as discussed in the next section.) Therefore, for the microwave frequency ^{87}Rb and ^{133}Cs clocks on board the GPS satellites, the sensitivity coefficients are[49,50]

$$\frac{\delta\omega}{\omega_c}(\mathrm{Rb}) = \varphi^2(4.34\,\Gamma_\alpha - 0.019\,\Gamma_q + \Gamma_{e/p}), \tag{12}$$

$$\frac{\delta\omega}{\omega_c}(\mathrm{Cs}) = \varphi^2(4.83\,\Gamma_\alpha + 0.002\,\Gamma_q + \Gamma_{e/p}), \tag{13}$$

respectively, where we have defined the short-hand notation $\Gamma_{e/p} \equiv 2\Gamma_{m_e} - \Gamma_{m_p}$, and $\Gamma_q \equiv \Gamma_{m_q/\Lambda_{\mathrm{QCD}}}$ (and similarly for $\Lambda_{e/p}$ and Λ_q). Note that the value of K_q comes from a combination of a shift in the nuclear magnetic moment, K_μ and a shift in the nuclear size, K_{hq}. For Cs, the contributions from these two effects are roughly equal in magnitude and opposite in sign[50] ($K_q^{\mathrm{Cs}} = K_\mu + K_{\mathrm{hq}} = 0.009 - 0.007$), and K_q in Cs is therefore sensitive to uncertainties in the nuclear calculations. For clocks based on optical transitions, there is only sensitivity to variations in the fine structure constant[46,47]. For clocks that compare the clock frequency to the resonant frequency of an optical cavity, there is an extra factor of α that comes from the variation in the length of the cavity, e.g.,

$$\frac{\delta\omega}{\omega_c}(\mathrm{Sr}_{\mathrm{optical}}) = \varphi^2\,1.06\,\Gamma_\alpha, \tag{14}$$

as in Ref. 51. In general, the cavity contributes no extra sensitivity to variations in m_e/m_p or $m_q/\Lambda_{\mathrm{QCD}}$[42,43].

The fact that there are a number of different atomic clock types employed within the GPS network means we have sensitivity to several different combinations of the parameters (9). The existing limits come from constraints on supernova emission[25]: $\Lambda_{m_e,\alpha} \gtrsim 3\,\mathrm{TeV}$, and $\Lambda_{m_p} \gtrsim 15\,\mathrm{TeV}$, with no existing limit on Λ_q.

Supplementary Note 3

Domain wall signal

One specific example of a TD is a domain wall, a quasi-2D structure that can be characterised by a width, d, and DM field amplitude[8], $\varphi_{\max}$ (see Fig. 1 of main text). We assume a Gaussian distribution of the field across the wall. As a wall passes a clock, it causes a shift in the clock frequency $\omega_c \to \omega_c + \delta\omega(t)$, where $\delta\omega(t)$ is proportional to the square of the field value and rapidly goes to zero outside of the wall. Then, the time-difference due to a transient frequency shift with respect to an unperturbed clock is $\Delta t = \int_{-\infty}^{t} \frac{\delta\omega(t')}{\omega_c} dt'$. The accumulated time-reading bias of clock i (measured against some reference clock, R) at time t caused by a Gaussian-profile domain-wall that crosses the clock at time $t_i^\times$ and the reference clock at $t_R^\times$, is

$$S_i^{(0)}(t) = \varphi_{\max}^2 \int_{-\infty}^{t} \left[\Gamma_{\mathrm{eff},i}\, \exp\left(\frac{-(t'-t_i^\times)^2}{\tau^2}\right) - \Gamma_{\mathrm{eff},R}\, \exp\left(\frac{-(t'-t_R^\times)^2}{\tau^2}\right)\right] dt', \tag{15}$$

where the (single-clock) crossing duration is defined as

$$\tau \equiv \frac{d}{v_\perp}, \tag{16}$$

which is the time scale during which the clock is inside the wall, and $v_\perp$ is the component of the wall's velocity that is perpendicular to the wall.

As mentioned above, to analyse the data, we first perform a single-differencing procedure (1). We perform the same differencing procedure for the DM signals. The correspondence between the $S^{(0)}$ and $S^{(1)}$ signals is shown in Supplementary Figure 3.

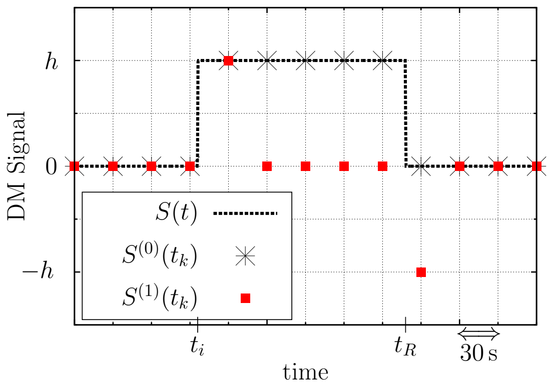

Supplementary Figure 3: Example of an ideal thin-wall signal for a pair of identical clocks separated by a distance $l = v(t_R - t_i)$, where the wall crosses the first clock at time t_i, and the second (reference) clock at time t_R. As the wall passes the first clock, it causes a shift in the clock frequency $\omega_c \to \omega_c + \delta\omega(t)$, where $\delta\omega(t)$ is only non-zero while the wall encompasses the clock. This leads to a difference in the time-readings (bias) between the two clocks, with magnitude $h \approx \delta\omega(t_i)\,\tau$, where $\tau = d/v_\perp$ is the interaction time. When the wall crosses the second clock, it causes the same frequency shift, so that after the wall has crossed, the time-reading difference returns to zero. The magnitude of the time-difference depends on the dark-matter–standard-model coupling strength, the speed of the wall (i.e. the duration of the interaction), and the width of the wall. The red squares correspond to the signal after applying the differencing technique, Supplementary Equation (1).

For thin walls (that is, for walls thin enough such that the interaction time is smaller than the data acquisition interval, $\tau < 30\,\mathrm{s}$), the $S^{(1)}$ spike amplitude would see in the first-order differenced data at the time of the wall crossing (for a system of identical clocks) is

$$S^{(1)} = \varphi_{\max}^2\,\Gamma_{\rm eff}\,\sqrt{\pi}\,\tau, \tag{17}$$

as shown in Supplementary Figure 3. Note that in the thin wall case, the assumed Gaussian profile is unimportant; other profiles (e.g., hard spheres, flat profiles) give similar results. These non-Gaussian profiles can be incorporated through the form factors in Supplementary Equation (17) arising from the integrals in Supplementary Equation (15). Our following analysis holds equally in these cases, the difference being only the ratio of the specific form factors. For example, for a flat (hard-edge) profile, $\Gamma_{\rm eff} \to \Gamma_{\rm eff}/\sqrt{\pi}$ in Supplementary Equation (17).

In order to determine the expected average $S^{(1)}$, we use the average value for the single-clock crossing duration, $\tau_{\rm avg}$, which depends on the object width d, orientation, and velocity distribution, as will be discussed in the Methods section of the main text. Further, in the assumption that the TDs saturate the DM energy density in the galaxy, one can link $\varphi_{\max}$ and d to $\rho_{\rm DM}$:

$$\varphi_{\max}^2 = \hbar c \rho_{\rm DM} d^2 \frac{\mathcal{T}}{\tau_{\rm avg}}, \tag{18}$$

where $\mathcal{T}$ is the average time between events (i.e., encounters between the GPS constellation and DM objects). Therefore, the average DM signal amplitude is:

$$S^{(1)}_{\rm avg} = \hbar c\,\Gamma_{\rm eff}\,\sqrt{\pi}\rho_{\rm DM}\,d^2\,\mathcal{T}. \tag{19}$$

This equation translates into constraints on $\Gamma_{\rm eff}$, Eq. (5) of the main text.

Supplementary Note 4

Search

Here, we perform a search with the aim of ruling out or detecting large ($\gtrsim 5\sigma$) events (here, σ is the standard deviation of $S^{(1)}$ clock data, which is about $0.1\,\mathrm{ns}$ in the worst-case for the Rb clock solutions when using another Rb clock as a reference, see Supplementary Table 1). Consider a time-period window, denoted J_w. If a thin-wall TD-DM object passes through the GPS constellation with a speed

$$v > D_{\mathrm{GPS}}/J_w, \tag{20}$$

where $D_{\mathrm{GPS}} \approx 50\,000\,\mathrm{km}$ is the GPS orbital diameter, we can expect that all of the clocks will be affected by the object within this window. However, any clock that is swept within the same 30 s sampling interval as the reference clock will not show any spike in the $S^{(1)}$ data due to clock degeneracy (see Supplementary Figure 3, or satellites 15 and 16 in Fig. 3(a) of the main text). For a DM object with relative velocity $v < 700\,\mathrm{km\,s^{-1}}$, we can safely expect at least 60% of the clocks to show an $S^{(1)}$ spike (this number comes from the orbital geometry of the GPS constellation, noting that by design the satellites are relatively evenly distributed over the sky). For more typical velocities, $v \sim 100 - 300\,\mathrm{km\,s^{-1}}$, more than 80% of the satellites are expected to show the $S^{(1)}$ spikes.

To ensure that the potential DM-induced frequency excursions for each clock are of the same magnitude, we only consider sub-networks of identical clocks. For example, for the Rb sub-network, we choose the most recently launched Rb satellite to be the reference clock (as a rule such clocks are the least noisy), and compare all the other Rb clocks to this reference, and do not include the Cs or H-maser clocks in the analysis. To unfold limits on the various coupling constants in Supplementary Equations (12) and (13), we only consider the Rb and Cs sub-networks, and supplement them with the Sr limits on Γ_α from Ref. 51. We do not include the ground clocks in our current analysis.

To search for domain wall signals, we analysed the $S^{(1)}$ GPS data streams in two stages. The first stage involved stepping through all the data, one epoch at a time, to identify regions of interest that could potentially be consistent with thin-wall crossings. We call these regions potential events. The second stage investigates these regions using a more detailed approach, to determine if we can upgrade any potential events to candidate events.

At the first stage, we scanned all the data from May 2000 to October 2016 looking for the most general patterns associated with a domain wall crossing, without taking into account the order in which the satellites were swept. We required at least 60% of the clocks to experience a frequency excursion at the same epoch, as discussed above. This procedure identifies the epoch when the wall crossed the reference clock (vertical blue line in Fig. 3(a) of the main text). If such a frequency excursion is found at epoch t_0, we form all the possible windows, J_w, around t_0, in the range $3 \leq J_w/30\,\mathrm{s} \leq 500$. The chosen maximum J_w determines the minimum DM velocity we can detect (20). Our choice of a maximum window of $500\,\mathrm{epochs}$, corresponding to sweep durations through the GPS constellation of up to 15,000 s, ensures sensitivity to walls moving as slowly as $\sim 4\,\mathrm{km\,s^{-1}}$. Less than 0.1% of DM domain walls that cross the GPS network are expected to travel with perpendicular velocities slower than this value.

We define a potential event as any time where at least the given fraction of the clocks also experience a frequency excursion of sign opposite to the reference clock excursion anywhere within that window (red tiles in Fig. 3 of main text). Since we consider only the sub-networks of identical clocks (e.g., we only consider the Rb clocks), if an event occurs within the window all the DM-induced frequency excursions must be of the same magnitude (besides the clock noise). Therefore, we only search for frequency excursions within a range of $S^{(1)}$ values, $S^{(1)}_{\mathrm{cut}} \to S^{(1)}_{\mathrm{cut}} \pm dS$, where $dS = x\sigma$, with σ being the calculated standard deviation for that given GPS clock/reference clock combination (see Supplementary Table 1); for 90% confidence level (C.L.), $x = 1.645$. By using the calculated standard deviation here, we account for both the formal error (see

Supplementary Table 2: An illustrative subset of the results of the stage 1 analysis, showing the number of potential events for the given cut-off values $S^{(1)}_{\rm cut}$ (using a 90% C.L. range) for the Rb satellite Global Positioning System sub-network for various time window lengths J_w. $S^{(1)}_{\rm thresh}$ is defined as the smallest $S^{(1)}_{\rm cut}$ value with zero potential events. The analysis covers a total effective period of 16.5 years, and includes a total of 131 353 clock-days of data.

	J_w (s)						
$S^{(1)}_{\rm cut}$ (ns)	90	270	450	630	810	990	1170
0.1	4295864	4387849	4387849	4387849	4387849	4387849	4387849
0.15	682150	973116	973133	973133	973133	973133	973133
0.2	69619	210761	211120	211127	211127	211127	211127
0.25	4969	46234	47214	47310	47322	47328	47330
0.3	363	10233	11727	11906	11955	11974	11994
0.35	23	1331	2290	2594	2680	2724	2737
0.4	1	97	235	345	385	413	437
0.45	0	5	20	29	39	45	51
0.5	0	1	3	4	4	5	5
0.55	0	0	1	1	1	1	1
0.6	0	0	0	1	1	1	1
0.65	0	0	0	0	0	0	0

Supplementary Note 1) and the clock noise directly. We count the total number of potential events for each window-size and $S^{(1)}_{\rm cut}$ combination.

This method also allows us to place more stringent limits on events for which the average time between events, $\mathcal{T}$, is less than the observation time, $\mathcal{T}_{\rm obs}$. For example, if for a given $S^{(1)}_{\rm cut}$, we saw 10 potential events in a 10 year observation time, we can place a limit at this level for $\mathcal{T} \simeq 1\,{\rm yr}$. Further, we only include days in our analysis for which there is data available for at least 7 clocks; this effectively reduced our observation time, especially for the Cs sub-network, which employs only a small number of clocks in recent times. Note, the choice of 7 simultaneously operational clocks here is conservative; injecting fake DM events demonstrates that this search technique would find practically every event (that otherwise falls within our current sensitivity) with at least 5 available clocks.

To perform the analysis, we first defined a signal cut-off $S^{(1)}_{\rm cut}$, see Supplementary Figure 2. We then systematically decreased the cut-off values and repeated the above analysis as depicted in Fig. 3 of the main text. Wherever the counted number of potential events goes to zero gives the overall limit for the total observation time, which we denote $S^{(1)}_{\rm thresh}$. For example, for a window size encompassing sweeps at $v \sim 300\,{\rm km\,s^{-1}}$ ($J_w = 170\,{\rm s}$), we can exclude events in the Rb network above the $S^{(1)}_{\rm thresh} = 0.48\,{\rm ns}$ level (90% C.L.), for a total observation time of $\mathcal{T}_{\rm obs} = 16.5\,{\rm yrs}$. Below the thresholds, a number of potential events were identified. An illustrative subset of the counted potential events is presented in Supplementary Table 2 for the Rb sub-network, and in Supplementary Table 3 for the Cs sub-network.

The second stage of the search involved analysing the potential events in more detail, and is described in the main text. While the detailed second stage analysis have improved (lowered) the values of $S^{(1)}_{\rm thresh}$ by rejecting a few hundred potential events, the improvement was minor. Analysing numerous potential events well below $S^{(1)}_{\rm thresh}$ (see Supplementary Table 2 and Supplementary Table 3) has proven to be substantially more computationally demanding, and is beyond the scope of the current work. Therefore, to be conservative, we have used the $S^{(1)}_{\rm thresh}$ results from the first stage of the analysis to place our constraints on DM walls.

Supplementary Table 3: As in Supplementary Table 2, but for the Cs Global Positioning System sub-network. Here, the analysis covers a total effective period of 10.5 years, and includes a total of 37 099 clock-days of data.

	J_w (s)						
$S^{(1)}_{\rm cut}$ (ns)	90	270	450	630	810	990	1170
0.1	6807411	6885078	6885078	6885078	6885078	6885078	6885078
0.15	2283645	3123230	3123326	3123326	3123326	3123326	3123326
0.2	476928	1490303	1492203	1492225	1492225	1492225	1492225
0.25	60719	552654	566282	566538	566555	566559	566559
0.3	6525	148529	171379	173184	173369	173395	173402
0.35	512	28240	40620	43181	43822	43993	44050
0.4	39	4264	7660	8982	9513	9742	9845
0.45	3	495	1087	1442	1655	1777	1883
0.5	0	52	125	182	222	257	285
0.55	0	8	16	24	28	33	41
0.6	0	1	2	3	3	4	4
0.65	0	1	1	1	1	1	1
0.7	0	0	0	0	0	0	0

Supplementary Note 5

Placing limits

The most stringent limit on the effective energy scale $\Lambda_{\rm eff} = 1/\sqrt{|\Gamma_{\rm eff}|}$ is set by assuming one event could have occurred in the observation time, which caused a frequency excursion in the timing data that was equal in magnitude to $S^{(1)}_{\rm thresh}$. In this case:

$$\mathcal{T} \to \mathcal{T}_{\rm obs}, \tag{21}$$

$$S^{(1)}_{\rm avg} \to S^{(1)}_{\rm thresh}. \tag{22}$$

To determine the confidence level for our limits, we must also factor in the uncertainties. The uncertainty from the clock noise is directly built into the $S^{(1)}$ values in Supplementary Table 2 and Supplementary Table 3. For our current analysis, the $S^{(1)}$ limits are substantially greater than the actual clock-noise, on the order of $5-20\sigma$. The dominating uncertainty in our limits comes from equating the observation time with the average time between events.

To set the limits in the region where $\mathcal{T} \simeq \mathcal{T}_{\rm obs}$, we assume that the frequency of DM–GPS encounters is roughly Poissonian. Suppose we expect to see on average ν events in the observation time $\mathcal{T}_{\rm obs}$. The probability for observing at least one event in the time period $\mathcal{T}_{\rm obs}$ is given by

$$P_{k\geq 1}(\nu) = 1 - P_0(\nu) = 1 - e^{-\nu}, \tag{23}$$

where $P_k(\nu) = \nu^k e^{-\nu}/k!$ is the Poisson distribution. For example, to place 90% confidence level limits we require that $P_{k\geq 1}(\nu) = 0.9$. In this case, solving (23) gives $\nu = 2.3$. Therefore, the maximum $\mathcal{T}$ for which we can place 90% C.L. limits is given by

$$\mathcal{T}_{\rm max} = \mathcal{T}_{\rm obs}/\nu, \tag{24}$$

where for a 90% C.L. limit, $\nu = 2.3$ and $\mathcal{T}_{\rm max} \simeq 7\,{\rm yr}$.

For the region of parameter space where $\mathcal{T} \ll \mathcal{T}_{\rm obs}$, our sensitivity is lower. This is because for small values of the average time between events, the total number density of DM objects must be correspondingly high, leading to smaller DM field values inside a defect. In the assumption that such objects constitute a

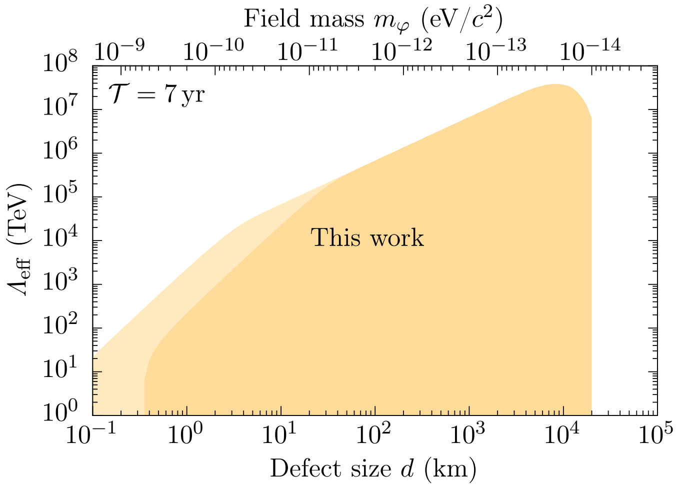

Supplementary Figure 4: Limits (90% C.L.) on the effective energy scale $\Lambda_{\rm eff}$ for Rb as a function of the wall width, d, for a fixed $\mathcal{T} = 7\,\mathrm{yr}$. The lighter and darker shaded yellow exclusion regions corresponding to the best and worst case scenarios, as described in the Methods section of the main text. Similar (but less stringent) limits can also be placed using the Cs sub-network. The kinks in the plot and the sharp cut off above $d \approx 2\times10^4\,\mathrm{km}$ are due to the crossing duration sensitivities, see Eqs. (11) and (14) of the main text. See also the contour plot in Fig. 4 of the main text.

significant fraction of the DM, their total energy must still add up to the total observed local DM density[52] of $\rho_{\rm DM} \approx 0.4\,\mathrm{GeV\,cm^{-3}}$:

$$\rho_{\rm inside} = \rho_{\rm DM}\frac{\mathcal{T} v_g}{d}. \tag{25}$$

Therefore, the energy density of each object must be smaller, and as such, the resulting signal would be smaller.

For the applicable region ($\mathcal{T} < \mathcal{T}_{\rm obs}/\nu$), we combine the $s(d)$ function with (19) to get the final constraints:

$$\frac{\Lambda_{\rm eff}}{d} > \sqrt{\frac{\hbar c\sqrt{\pi}\rho_{\rm DM}\,\mathcal{T}\,s(d)}{S^{(1)}_{\rm thresh}}}. \tag{26}$$

Similarly, in the case when one of the specific couplings, Γ_X, dominates over the other coupling strengths in the linear combination in Supplementary Equations (12) and (13), we have

$$\frac{\Lambda_X}{d} > \sqrt{\frac{\hbar c\sqrt{\pi}\rho_{\rm DM}\,\mathcal{T}\,s(d)\,K_X}{S^{(1)}_{\rm thresh}}}. \tag{27}$$

Note that the only d-dependence in $s(d)$ comes from the integration limits (via $\tau_{\rm min/max}$). Expressing in convenient units:

$$\frac{\Lambda_{\rm eff}/\mathrm{TeV}}{d/\mathrm{km}} > 2\times10^3\sqrt{\frac{s(d)\,\mathcal{T}/\mathrm{yr}}{S^{(1)}_{\rm thresh}/\mathrm{ns}}}. \tag{28}$$

Using the relation $d = \hbar/m_\varphi c$, one can rewrite the above limit in terms of the field mass m_φ with the substitution $(d/\mathrm{km}) \approx 2\times10^{-10}\,(\mathrm{eV}/m_\varphi c^2)$.

The resulting 90% C.L. limits on $\Lambda_{\rm eff}$ from combining the $S^{(1)}$ limits from Supplementary Table 2 and Supplementary Table 3 with Supplementary Equation (26), for the case when $\mathcal{T} = \mathcal{T}_{\rm obs}/\nu = 7\,\mathrm{yr}$, are shown in Supplementary Figure 4. For smaller values of $\mathcal{T}$, the limits scale as $\sqrt{\mathcal{T}}$. See also Fig. 4 of the main text, which shows a contour plot of the constraints as a function of d and $\mathcal{T}$.

Recently, the group from Toruń[51] used an optical Sr clock to place limits on the coupling of topological defect DM to atoms. Since this group employed an optical transition in Sr, this experiment is only sensitive

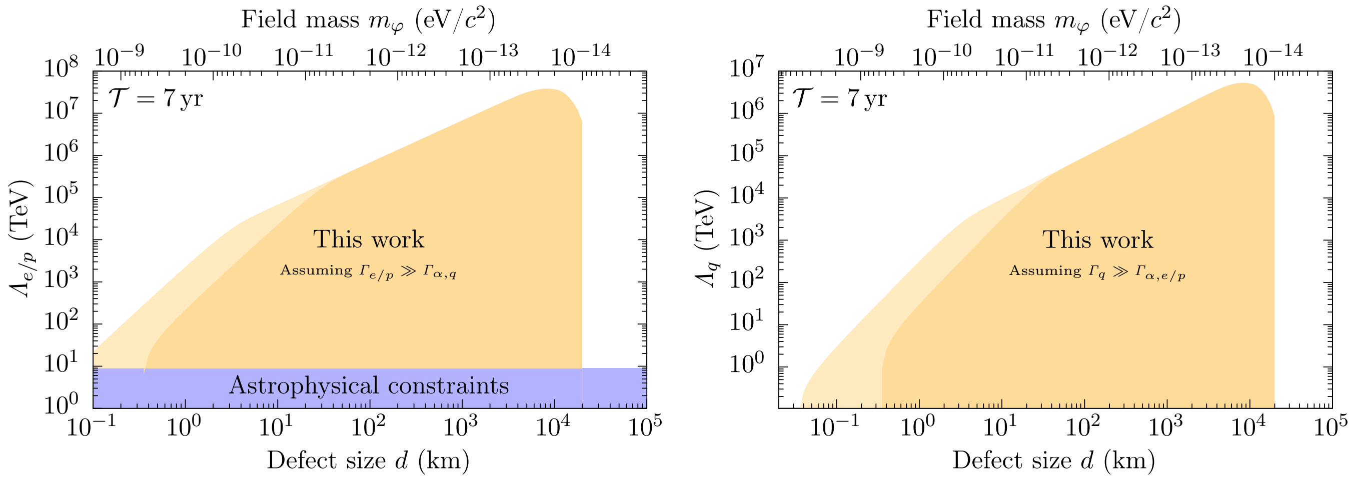

Supplementary Figure 5: Limits (90% C.L.) on individual energy scales from the Rb sub-network, assuming these respective couplings dominate the linear combination in Supplementary Equation (12). Limits are shown as a function of the wall width, d, with fixed $\mathcal{T} = 7\,\text{yr}$, for $\Lambda_{e/p}$ (left panel) and Λ_q (right panel). These exclusion regions are shown in yellow, with the lighter and darker regions as in Supplementary Figure 4. Existing astrophysical bounds[25] are shown in blue; note that Λ_q was previously unconstrained.

to the variation in the fine structure constant (i.e., Λ_α), see Supplementary Equation (14). Their data covers a period of $\mathcal{T}_{\text{obs}} = 45700\,\text{s} \approx 13\,\text{hrs}$. We combine their derived limits on Λ_α with our results in the exclusion plots. We note that although tight constraints can be placed using this method[51], in order to distinguish a true DM-induced transient event from other external sources (such as electromagnetic interference, or direct physical disturbance of the clocks), a global network is prerequisite . One of the main advantages of our method of employing the GPS constellation is the reliance on such a global network. Another advantage of our approach is the availability of archival data for at least the past 16 years, giving us sensitivity to the region of the parameter space with $\mathcal{T} \gtrsim 1 - 10\,\text{yrs}$, which is currently inaccessible by other methods.

If we make assumptions about the relative strengths of the couplings in Supplementary Equation (9), we can place limits on individual energy scales (27). For example, in the assumption that $\Lambda_\alpha \ll \Lambda_{e/p}, \Lambda_q$, we can place limits directly on Λ_α (and likewise for $\Lambda_{e/p}$ and Λ_q). These resulting limits for Λ_α (and comparison with the results of Ref. 51) are shown in Fig. 5 of the main text. Note that due to differences in the experimental technique, the optical Sr limits scale with the wall width as $d^{3/4}$, and the sensitivity of the approach of Ref. 51 reduces sharply for widths greater than the Earth radius due to a frequency cut-off used in the analysis[51]. In contrast, the GPS limits from this work scale linearly with d (27). Both our limits and those of Ref. 51 scale as $\sqrt{\mathcal{T}}$, but have sharp cut-offs above the observation time ($\mathcal{T}_{\text{obs}} \sim 16\,\text{yr}$ for our work, and $\mathcal{T}_{\text{obs}} \sim 10^{-3}\,\text{yr}$ for Ref. 51). Limits on $\Lambda_{e/p}$ and Λ_q (assuming these respective couplings dominate) are shown in Supplementary Figure 5 (these couplings are unconstrained by the Sr experiment[51]).

From the three independent limits (one from each of the Rb and Cs sub-networks determined in this work, and one from the optical Sr clock used in Ref. 51), we can derive independent limits on $\Lambda_{e/p}$ and Λ_q, without having to make assumptions about the relative strengths of the individual couplings. This is possible because the three limits (from Cs, Rb, and Sr) depends on a different linear combination of the three available couplings, see Supplementary Equations (12)–(14). A plot showing the combined allowed region for the coupling parameters Γ_α, $\Gamma_{e/p}$, and Γ_q is presented in Supplementary Figure 6 (note $\Lambda_X = 1/\sqrt{|\Gamma_X|}$). The resulting limits on $\Lambda_{e/p}$ and Λ_q are presented in Figs. Supplementary Figure 7 and Supplementary Figure 8, respectively.

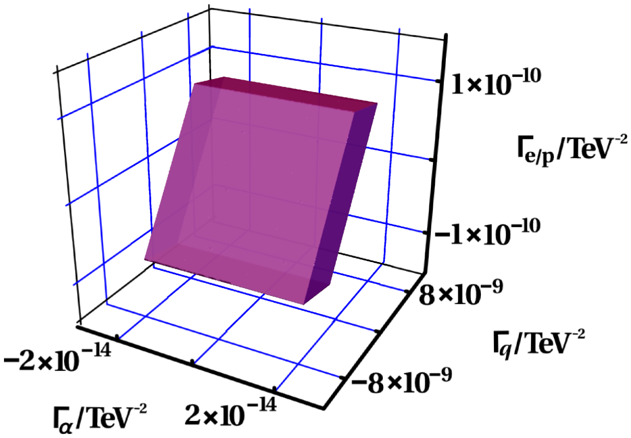

Supplementary Figure 6: Allowed region for the coupling strength parameters Γ_α, $\Gamma_{e/p}$, and Γ_q, from the combined limits using the Rb and Cs Global Positioning System sub-networks, and the Sr optical clock limits from Ref. 51, for fixed $d = 10^3$ km and $\mathcal{T} = 10^{-3}$ yr. Note that the allowed region (the inner part of the parallelepiped, shown in purple) is completely bound on all sides.

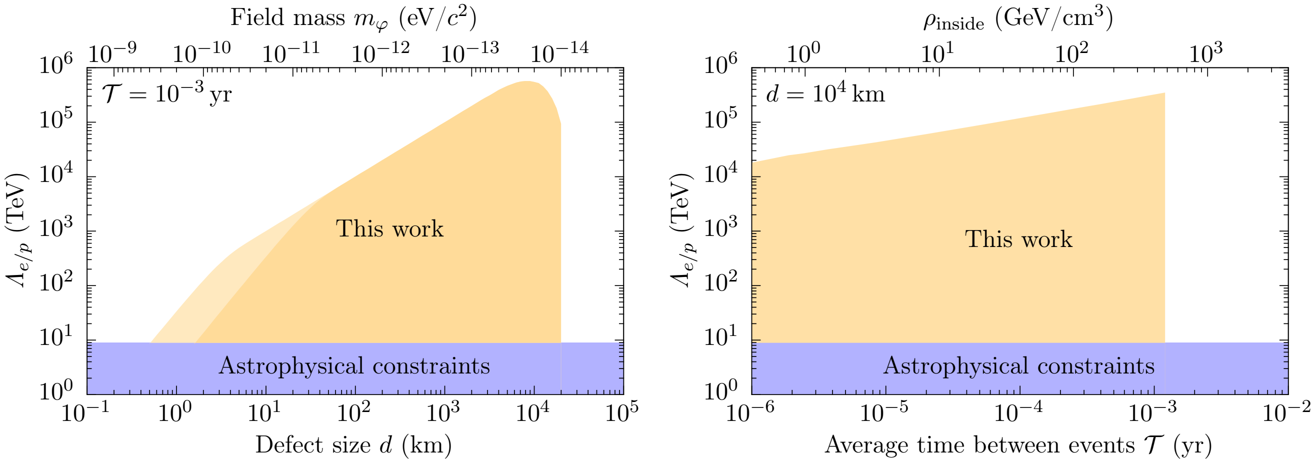

Supplementary Figure 7: Limits on $\Lambda_{e/p}$ (90% C.L.), by combining the limits from the Rb and Cs Global Positioning System clocks (this work) and the limits from the optical Sr clock from Ref. 51, making no assumptions on the relative coupling strengths. Shown as a function of d, with fixed $\mathcal{T} = 45700$ s, and as a function of $\mathcal{T}$, with fixed $d = 10^4$ km.

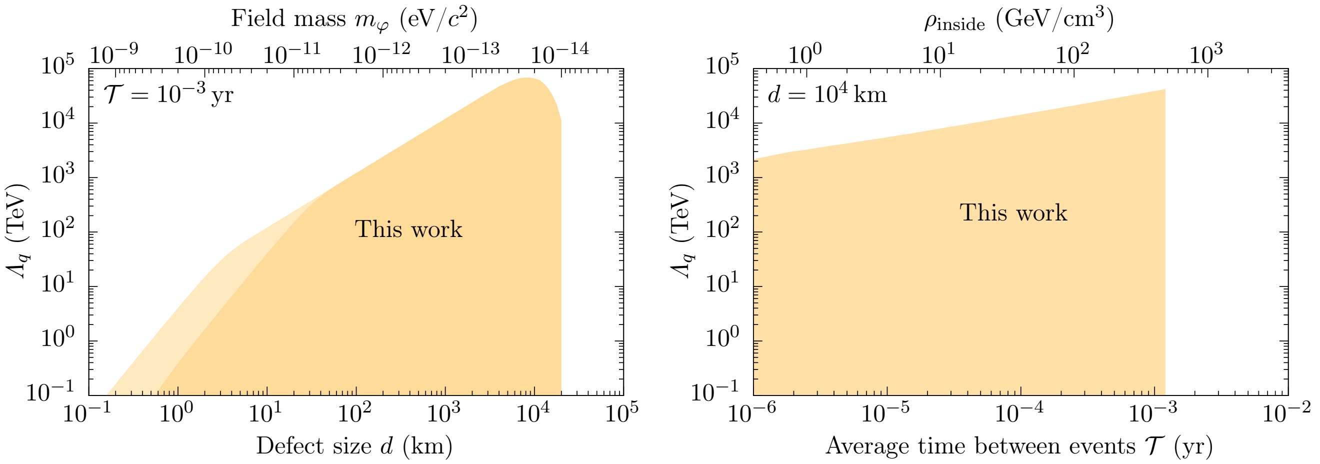

Supplementary Figure 8: Limits on Λ_q (90% C.L.), by combining the limits from the Rb and Cs Global Positioning System clocks (this work) and the limits from the optical Sr clock from Ref. 51, making no assumptions on the relative coupling strengths. Shown as a function of d, with fixed $\mathcal{T} = 45700\,\mathrm{s} \approx 10^{-3}\,\mathrm{yr}$, and as a function of $\mathcal{T}$, with fixed $d = 10^4\,\mathrm{km}$.

Supplementary References

[1] Blewitt, G. Carrier phase ambiguity resolution for the Global Positioning System applied to geodetic baselines up to 2000 km. *J. Geophys. Res. Solid Earth* **94**, 10187–10203 (1989).

[2] Blewitt, G. An Automatic Editing Algorithm for GPS data. *Geophys. Res. Lett.* **17**, 199–202 (1990).

[3] Jet Propulsion Laboratory. ftp://sideshow.jpl.nasa.gov/pub/jpligsac/.

[4] D. Murphy *et al.* JPL Analysis Center Technical Report *(in IGS Technical Report)* **2015**, 77 (2015).

[5] GPS.gov. http://www.gps.gov/systems/gps/space/.

[6] The International GNSS Service (IGS). http://www.igs.org/network.

[7] Budker, D. & Derevianko, A. A data archive for storing precision measurements. *Phys. Today* **68**, 10–11 (2015).

[8] Derevianko, A. & Pospelov, M. Hunting for topological dark matter with atomic clocks. *Nat. Phys.* **10**, 933–936 (2014).

[9] Pospelov, M. *et al.* Detecting domain walls of axionlike models using terrestrial experiments. *Phys. Rev. Lett.* **110**, 021803 (2013).

[10] Pustelny, S. *et al.* The Global Network of Optical Magnetometers for Exotic physics (GNOME): A novel scheme to search for physics beyond the Standard Model. *Ann. Phys.* **525**, 659–670 (2013).

[11] Peccei, R. D. & Quinn, H. R. CP Conservation in the Presence of Pseudoparticles. *Phys. Rev. Lett.* **38**, 1440–1443 (1977).

[12] Peccei, R. D. & Quinn, H. R. Constraints imposed by CP conservation in the presence of pseudoparticles. *Phys. Rev. D* **16**, 1791–1797 (1977).

[13] Dine, M., Fischler, W. & Srednicki, M. A simple solution to the strong CP problem with a harmless axion. *Phys. Lett. B* **104**, 199–202 (1981).

[14] Sikivie, P. Experimental tests of the "invisible" axion. *Phys. Rev. Lett.* **51**, 1415–1417 (1983).

[15] Preskill, J., Wise, M. B. & Wilczek, F. Cosmology of the invisible axion. *Phys. Lett. B* **120**, 127–132 (1983).

[16] Sikivie, P. Axions, domain walls, and the early universe. *Phys. Rev. Lett.* **48**, 1156–1159 (1982).

[17] Press, W. H., Ryden, B. S. & Spergel, D. N. Dynamical evolution of domain walls in an expanding universe. *Astrophys. J.* **347**, 590 (1989).

[18] Vilenkin, A. & Shellard, E. *Cosmic Strings and Other Topological Defects* (Cambridge University Press, Cambridge, 1994).

[19] Battye, R. A., Bucher, M. & Spergel, D. N. Domain wall dominated universes. Preprint at http://arxiv.org/abs/astro-ph/9908047 (1999).

[20] Durrer, R., Kunz, M. & Melchiorri, A. Cosmic structure formation with topological defects. *Phys. Rep.* **364**, 1–81 (2002).

[21] Friedland, A., Murayama, H. & Perelstein, M. Domain walls as dark energy. *Phys. Rev. D* **67**, 043519 (2003).

[22] Avelino, P. P., Martins, C. J. A. P., Menezes, J., Menezes, R. & Oliveira, J. C. R. E. Dynamics of domain wall networks with junctions. *Phys. Rev. D* **78**, 103508 (2008).

[23] Raffelt, G. G. Particle physics from stars. *Annu. Rev. Nucl. Part. Sci.* **49**, 163–216 (1999).

[24] Bertotti, B., Iess, L. & Tortora, P. A test of general relativity using radio links with the Cassini spacecraft. *Nature* **425**, 374–376 (2003).

[25] Olive, K. A. & Pospelov, M. Environmental dependence of masses and coupling constants. *Phys. Rev. D* **77**, 043524 (2008).

[26] Coleman, S. Q-balls. *Nucl. Phys. B* **262**, 263–283 (1985).

[27] Dvali, G., Kusenko, A. & Shaposhnikov, M. New physics in a nutshell, or Q-ball as a power plant. *Phys. Lett. B* **417**, 99–106 (1998).

[28] Kusenko, A. & Steinhardt, P. J. Q-ball candidates for self-interacting dark matter. *Phys. Rev. Lett.* **87**, 141301 (2001).

[29] Hogan, C. J. & Rees, M. J. Gravitational interactions of cosmic strings. *Nature* **311**, 109–114 (1984).

[30] Marsh, D. J. E. & Pop, A.-R. Axion dark matter, solitons and the cusp-core problem. *Mon. Not. R. Astron. Soc.* **451**, 2479–2492 (2015).

[31] Kusenko, A. Solitons in the supersymmetric extensions of the standard model. *Phys. Lett. B* **405**, 108–113 (1997).

[32] Lee, T. D. & Pang, Y. Nontopological solitons. *Phys. Rep.* **221**, 251–350 (1992).

[33] Hogan, C. & Rees, M. Axion miniclusters. *Phys. Lett. B* **205**, 228–230 (1988).

[34] Kolb, E. W. & Tkachev, I. I. Axion miniclusters and Bose stars. *Phys. Rev. Lett.* **71**, 3051–3054 (1993).

[35] Jetzer, P. Boson stars. *Phys. Rep.* **220**, 163–227 (1992).

[36] Budker, D., Graham, P. W., Ledbetter, M. P., Rajendran, S. & Sushkov, A. O. Proposal for a Cosmic Axion Spin Precession Experiment (CASPEr). *Phys. Rev. X* **4**, 021030 (2014).

[37] Stadnik, Y. V. & Flambaum, V. V. Searching for topological defect dark matter via nongravitational signatures. *Phys. Rev. Lett.* **113**, 151301 (2014).

[38] Arvanitaki, A., Huang, J. & Van Tilburg, K. Searching for dilaton dark matter with atomic clocks. *Phys. Rev. D* **91**, 015015 (2015).

[39] Arvanitaki, A., Dimopoulos, S. & Van Tilburg, K. Sound of dark matter: searching for light scalars with resonant-mass detectors. *Phys. Rev. Lett.* **116**, 031102 (2016).

[40] Stadnik, Y. V. & Flambaum, V. V. Can dark matter induce cosmological evolution of the fundamental constants of nature? *Phys. Rev. Lett.* **115**, 201301 (2015).

[41] Arvanitaki, A. & Geraci, A. A. Resonant detection of axion mediated forces with nuclear magnetic resonance. *Phys. Rev. Lett.* **113**, 161801 (2014).

[42] Stadnik, Y. V. & Flambaum, V. V. Enhanced effects of variation of the fundamental constants in laser interferometers and application to dark-matter detection. *Phys. Rev. A* **93**, 063630 (2016).

[43] Stadnik, Y. V. & Flambaum, V. V. Searching for dark matter and variation of fundamental constants with laser and maser interferometry. *Phys. Rev. Lett.* **114**, 161301 (2015).

[44] Arvanitaki, A., Graham, P. W., Hogan, J. M., Rajendran, S. & Van Tilburg, K. Search for light scalar dark matter

with atomic gravitational wave detectors. Preprint at http://arxiv.org/abs/1606.04541 (2016).

[45] Hall, E. D. *et al.* Laser interferometers as dark matter detectors. Preprint at http://arxiv.org/abs/1605.01103 (2016).

[46] Dzuba, V. A., Flambaum, V. V. & Marchenko, M. V. Relativistic effects in Sr, Dy, Yb II, and Yb III and search for variation of the fine-structure constant. *Phys. Rev. A* **68**, 022506 (2003).

[47] Angstmann, E. J., Dzuba, V. A. & Flambaum, V. V. Relativistic effects in two valence-electron atoms and ions and the search for variation of the fine-structure constant. *Phys. Rev. A* **70**, 014102 (2004).

[48] Dupuis, R. T., Lynch, T. J. & Vaccaro, J. R. Rubidium Frequency Standard for the GPS IIF program and modifications for the RAFSMOD Program. In *2008 IEEE Int. Freq. Control Symp.*, 655–660 (IEEE, 2008).

[49] Flambaum, V. V. & Tedesco, A. F. Dependence of nuclear magnetic moments on quark masses and limits on temporal variation of fundamental constants from atomic clock experiments. *Phys. Rev. C* **73**, 055501 (2006).

[50] Dinh, T. H., Dunning, A., Dzuba, V. A. & Flambaum, V. V. Sensitivity of hyperfine structure to nuclear radius and quark mass variation. *Phys. Rev. A* **79**, 054102 (2009).

[51] Wcisło, P. *et al.* Experimental constraint on dark matter detection with optical atomic clocks. *Nat. Astron.* **1**, 0009 (2016).

[52] Nesti, F. & Salucci, P. The Dark Matter halo of the Milky Way, AD 2013. *J. Cosmol. Astropart. Phys.* **2013**, 016–016 (2013).
